# Haematology, biochemistry and morphological features of peripheral blood cells in captive *Boa constrictor*

**DOI:** 10.1093/conphys/coad001

**Published:** 2023-01-28

**Authors:** E Dervas, E Michalopoulou, A Liesegang, M Novacco, F Schwarzenberger, U Hetzel, A Kipar

**Affiliations:** Institute of Veterinary Pathology, Vetsuisse Faculty, University of Zurich, Winterthurerstrasse 268, 8057, Zurich, Switzerland; Institute of Veterinary Pathology, Vetsuisse Faculty, University of Zurich, Winterthurerstrasse 268, 8057, Zurich, Switzerland; Institute for Animal Nutrition and Dietetics, Vetsuisse Faculty, University of Zurich, Winterthurerstrasse 270, 8057, Zurich, Switzerland; Clinical Laboratory, Vetsuisse Faculty, University of Zurich, Winterthurerstrasse 260, 8057, Zurich, Switzerland; Unit of Physiology, Pathophysiology and Experimental Endocrinology, Department of Biomedical Sciences, University of Veterinary Medicine Vienna, Veterinärplatz 1, 1210, Vienna, Austria; Institute of Veterinary Pathology, Vetsuisse Faculty, University of Zurich, Winterthurerstrasse 268, 8057, Zurich, Switzerland; Institute of Veterinary Pathology, Vetsuisse Faculty, University of Zurich, Winterthurerstrasse 268, 8057, Zurich, Switzerland

## Abstract

The common boa (*Boa constrictor)* belongs to the family Boidae and represents one of the most popular traded and kept snake species in captivity. The early diagnosis, prevention and prophylaxis of diseases in this species, and in reptiles in general, still pose major challenges, also due to the lack of reliable reference values. This prompted us to conduct a study on clinically healthy captive *B. constrictor* to assess their basic health parameters in the blood (haematological and biochemical values, stress markers). Several parameters differed significantly between younger (<3 years) and older (≥3 years) boas; in the latter, the percentages of eosinophils, the haemoglobin and haematocrit levels, as well as the albumin and total protein levels, were higher. In male snakes, cholesterol levels were significantly higher than in females. Light and electron microscopy as well as immunohistochemistry served to identify and determine the morphological features of peripheral blood cells, that is, heterophils, basophils, eosinophils, azurophils, monocytes, lymphocytes, thrombocytes and erythrocytes. Leukocyte subpopulations, that is, *T* and B cells and monocytes, were also identified based on specific marker expression. The study provides data on haematological, biochemical and stress hormone levels, suitable as reference values, and on the blood cell morphology of *B. constrictor* which can serve as a guideline for further research on this species.

## Teaser

Assessment of health and disease in reptiles is challenging as basic health parameters are not known for many reptile species. We undertook an in-depth study to determine basic blood parameters, stress hormone levels and the morphological features of peripheral blood cells in *Boa constrictor*, a common captive reptile species.

## Introduction

*Boa constrictor*, the sole species historically allocated to the monotypic genus *Boa* of the family *Boidae*, is a large non-venomous viviparous snake indigenous in tropical forests of Central and South America. The *B. constrictor* is one of the longest-lived snakes with a life span in captivity of 20 years or more (Divers, 1996). It is one of the snake species most frequently traded as well as kept and bred in captivity (Montgomery *et al.*, 2015). In most countries, the husbandry of *B. constrictor* follows defined guidelines that, despite being closely adapted to the natural environment, subject the animal to very ‘standardized’ housing conditions regarding environmental temperature and humidity, enclosure size etc., with only little seasonal variation (van Waeyenberge *et al.*, 2018). Captivity in general has often been stated to expose reptiles to stress, favouring the development of immunosuppression and, therefore, infectious diseases (Warwick *et al.*, 2013; van Waeyenberge *et al.*, 2018; Morgan and Tromborg, 2007; Schumacher, 2006). Another risk for infections comes from the mixing of species from different geographic regions, resulting in the potential exposure to pathogens that the animals have previously not been in contact with (Schumacher, 2006). *B. constrictor* are susceptible to a wide range of viral, bacterial and parasitic infections. Important viral agents reported in this species are reptarenaviruses (the causative agents of Boid inclusion body disease, BIBD) (Hetzel *et al.*, 2013) and the ophidian paramyxovirus (Piewbang *et al.* 2021). Parasitic infections are also of relevance; reported are, for example, arthropods (mites, e.g. *Ophionyssus natricis),* intestinal parasites (e.g. Cryptosporidia) and blood parasites *(Hepatozoon sp*.) (García-Márquez *et al*., 2019).

Although knowledge on appropriate maintenance and upbringing of captive snakes is growing, the early diagnosis, prevention and prophylaxis of infectious diseases still poses major challenges. In general, establishing a definitive clinical diagnosis is often difficult in reptiles, as the animals show only few pathognomonic clinical signs and often have a chronic disease course (Divers, 1996). In both human and veterinary medicine, haematological and biochemical parameters are important, widely used markers for assessment of the health status, clinical diagnosis and prognosis of disease, and to monitor the response to treatment. They are also fundamental tools to monitor the health status of wild and captive reptiles (Christopher *et al.*, 1999; Lisičić *et al.*, 2013). However, in contrast to domestic animals, haematological and biochemical data in reptiles are currently not as readily available because reference values are still lacking for most species (Nardini *et al.*, 2013). The latter may also be the reason why haematologic and biochemical values in reptiles are often interpreted in analogy to those of mammals (Grego *et al.*, 2006). Establishment of haematological reference intervals is also hardened by the inconsistent classification of leukocyte subpopulations in reptiles, as some authors characterized peripheral blood cells based on their function (Carvalho *et al.*, 2017), others solely based on their morphology (Svoboda *et al.*, 2006; Zimmerman *et al.*, 2010).

Similarly, the determination of stress in reptiles, particularly of chronic stress, also remains challenging. Corticosterone levels are commonly used as biomarker for stress in reptiles (van Waeyenberge *et al.*, 2018), yet the interpretation of the results is difficult as, similar to blood parameters, they are strongly dependent on several extrinsic parameters (eg. temperature, season, habitat, diet, disease, stress, venipuncture site) as well as intrinsic factors (species, sex, age, physiologic status) (Joseph, 2015).

Studies that provide reference values for haematological, biochemical and stress markers in boid snakes are sparse and focus mainly on wild animals and their seasonal variations (Fonseca Sarmiento *et al.*, 2018; Machado *et al.*, 2006; Quadrini *et al.*, 2018). The present study aimed to establish basic haematologic and biochemical parameters and to gather data on the ‘stress level’ of clinically healthy captive *B. constrictor*, to obtain a diagnostic tool set for the assessment of their health status.

## Material and Methods

**Animals.** The study was undertaken on blood samples collected from 79 clinically healthy *B. constrictor* originating from collections of Swiss (A, C-L) and German (M) breeders or shelters.

Twenty-one animals (B1-B21) originated from a reptile shelter that takes over reptiles from private owners or breeders who are unable to maintain their collection. The snakes were euthanized as the shelter had not succeeded in finding new owners for the animals within a prolonged period of time. Euthanasia was performed at the Institute of Veterinary Pathology, Vetsuisse Faculty, University of Zurich, followed by blood collection and a full diagnostic post mortem examination to exclude any disease and to obtain information on the general health status in the facility. Information on the exact origin of the snakes was not available.

In all animals, the blood sampling was undertaken upon the owners’ request and with their full consent, with the aim to diagnose or exclude BIBD and reptarenavirus infection, as a means to determine the infection status of the shelter and the collections, respectively. In live animals (A10-A28, C1-C7, D1-2), it was performed onsite in the collection. All blood sampling was undertaken on the basis of a project permit (cantonal number ZH 195/2016 and ZH136/2020) from the Cantonal Veterinary Office in Zurich.

In the cases where euthanasia was performed, an ASPA (Animals Scientific Procedures Act 1986) schedule 1 (appropriate methods of humane killing (http://www.legislation.gov.uk/ukpga/1986/14/schedule/1)) procedure was applied.

All animals were tested negative for BIBD by cytological examination of a blood smear (see below).

### Blood sampling

Blood samples were collected from living animals by cardiocentesis using a 22- or 25-gauge needle on a 3-ml syringe. The heart was visualized by a SonoTrax Vascular Doppler (FS15575, EDAN, USA). A blood volume of 1–2 ml was collected into heparinized tubes (Sarstedt, Germany), the volume depending on the size of each animal but not exceeding 5–8% of the total blood volume (Nardini *et al.*, 2013).

From dead animals, blood was extracted by cardiocentesis using a 22- or 25-gauge needle on a 3-ml syringe immediately after euthanasia.

### Blood cytology

Smears were prepared from all blood samples and stained with May–Grünwald–Giemsa. They were subjected to a cytological examination to identify the morphological features of leukocyte subpopulations and to determine the BIBD-negative status of the animals (no evidence of the pathognomonic cytoplasmic inclusion bodies (IB) within blood cells), as previously described (Hetzel *et al.*, 2013).

### Buffy coat preparation, processing and staining, immunohistochemistry, RNA-ISH and transmission electron microscopy

In 13 animals, the blood volume was sufficient to also prepare a buffy coat. Plain micro haematocrit capillary tubes (BR749321, Merck, Germany) were filled with blood and spun in a microhaemofuge (Heraeus Medical AG, Switzerland) at 12 700 *g* for 5 min according to previously described protocols (Fontes Pinto *et al.*, 2018). Tubes were broken at the plasma-buffy coat interface using a diamond pen, and the remaining portion of the capillary tubes (containing the buffy coat) immersed in either 4% formalin for 24 h for light microscopy, or 2.5% glutaraldehyde for 24 h and then buffered in 0.2 M cacodylic acid buffer, pH 7.3 for transmission electron microscopy (TEM).

The formalin-fixed buffy coat pellets were paraffin wax embedded. Consecutive sections (3–5 μm) were prepared, stained with haematoxylin eosin (HE) and subjected to RNA in situ-hybridization (RNA-ISH) and immunohistochemical staining, respectively.

Immunohistochemistry served for the identification of *T* cells (CD3+) and cells with monocytic morphology (monocytes and azurophils; Iba1+), applying cross-reacting antibodies and the horseradish peroxidase method, and using a Dako autostainer (Dako, Glostrup, Denmark). Briefly, after deparaffinization, antigen retrieval was performed by incubation of the slides with citrate buffer (pH 6) for Iba1 or EDTA buffer (pH 9) for CD3 at 98°C for 20 min in a pressure cooker. After incubation with the primary antibodies, rabbit anti-human Iba1 (1:350, 019–19 741, Wako, Osaka, Japan) overnight at 4°C and mouse anti-human CD3 (1:150, M725401, clone F7.2.38, Dako) for 1 h at room temperature (RT), sections were incubated with the secondary antibodies (EnVision+ HRP Rabbit (Dako) for anti-Iba1, EnVision+HRP Mouse (Dako) for anti-CD3) according to the manufacturer’s protocol. Endogenous peroxidase was blocked by incubation with peroxidase blocking solution (Dako) for 10 min at RT. Sections were washed with TBS buffered saline (pH 7) between each incubation step. Finally, sections were counterstained with haematoxylin for 20 s and mounted. Slides incubated with non-immune serum from the species in which the primary antibody was raised instead of the primary antibodies served as negative controls. Lymphoid tissue of *B. constrictor* served as a positive control.

For the identification of B cells, RNA-ISH was performed using the RNAscope® technology (Advanced Cell Diagnostics (ACD), Silicon Valley, USA) and the RNAscope 2.5 Detection Reagent Kit (Brown) according to the manufacturer’s protocol. All cases were first tested for the suitability of the material (RNA preservation and quality) with an oligoprobe for mouse ubiquitin (MUC) (BLAST analysis (NCBI, BLAST) of the MUC oligoprobe sequence to the *python bivitattus* genome (accession number XM_015890515.2) identified 87.99% as the highest percentage of identity). Those yielding good MUC signals were then subjected to RNA-ISH with oligoprobes coding for *B. constrictor* CD20 (mRNA Sequence ID XM_007431414.3). Briefly, sections were heated to 60°C for 1 h and subsequently deparaffinized. Permeabilization was achieved by incubation in pretreatment solution 1 (RNAscope Hydrogen Peroxide) for 10 min at RT. Afterwards, the sections were boiled in RNAscope 1× Target Retrieval Reagents solution at 100°C for 15 min, followed by washing in distilled water and ethanol. After digestion with RNAscope Protease Plus for 30 min at 40°C, sections were hybridized with the oligoprobes at 40°C in a humidity control tray for 2 h (HybEZ Oven, ACD). Thereafter, serial amplification with different amplifying solutions (AMP1, AMP2, AMP3, AMP4: alternating 15 and 30 min at 40°C) was performed. Between each incubation step, slides were washed with washing buffer. They were subsequently incubated with AMP5, AMP6 and DAB at RT for 30 min and 15 min, respectively. Gill’s haematoxylin served to counterstain the sections that were then dehydrated with graded alcohol and xylene and coverslipped. Consecutive sections incubated accordingly but without including the hybridization step served as negative controls.

For TEM, glutaraldehyde buffy coat pellets from 3 adult animals were routinely embedded in epoxy resin. Toluidine blue-stained semithin sections (1.5 μm) were prepared to select areas of interest for the preparation of ultrathin sections (75 nm). The latter were contrasted with lead citrate and uranyl acetate and viewed with a Philips CM10, operating with a Gatan Orius Sc1000 digital camera (Gatan Microscopical Suite, Digital Micrograph, Pleasanton, USA).

### Morphological identification of blood cells

For the identification of the blood cells and to determine their morphological features, previous literature was consulted. To identify the blood cells in cytologic smears, we referred to literature on other snake families/species, for example, kingsnakes, kobras, blood pythons and pine snakes (Salakij *et al.*, 2002; Giori *et al.*, 2020; Stacy *et al.*, 2011) and/or other reptile classes (Moller *et al.*, 2016) as data on the morphological features of blood cells in *B. constrictor* were not available. The ultrastructural identification of blood cells was also partially based on findings from previous studies on other reptile species (Salakij *et al.*, 2002; Moller *et al.*, 2016). However, there was no literature on reptiles that could be referred to for a clear differentiation and distinction of certain cell types, for example, azurophils and monocytes. Therefore, we used the ‘monocytic’ features known from mammals, that is, horses and dogs (Mehta and Sinha, 2018; Pereira *et al.*, 2019) to identify monocytes ultrastructurally. Azurophils were identified based on their appearance and overall high number in the cytological smear (Stacy *et al.*, 2011); this was then correlated with their location in the buffy coat and the expression of Iba1, a monocyte marker expressed across different orders (Pierezan *et al.*, 2014).

An overall distinction between blood cells of the granulocytic and agranulocytic lineage as applied in some former reptile studies (Hüseyin Arıkan, 2014; Jenkins-Perez, 2012) was not found to be appropriate for the present study. This was due to the fact that monocytes, historically belonging to the ‘agranulocytes’ (McMillan and Harris RJ, 2018), exhibited structures in TEM that were ultrastructurally consistent with intracytoplasmic granules; these have also been described in humans (Collin *et al.*, 2016).

### Haematology

Haematological parameters (total leukocyte count, haematocrit, haemoglobin, mean cell haemoglobin concentration (MCHC), heterophil, azurophil, lymphocyte, eosinophil, basophil and monocyte counts) were assessed in 49 individuals (Supplemental Table 1).

Blood smears were prepared and stained with an automated staining instrument (HEMA-TEK 2000 slide stainer, Bayer HealthCare AG, Berlin, Germany), using a modified Wright-Giemsa solution (Hematek® Stain Pak, Siemens Healthcare Inc., New York, USA) within 6 h of sampling and air dried. A 100 white blood cell (WBC) differential count was performed by two laboratory technicians with experience in reptilian haematology. Out of the two differential counts, the mean was calculated to obtain a percentage for each cell type.

The packed cell volume (PCV) was determined by placing blood into a plain glass microhaematocrit tube (Arnold Bott AG, Glattbrugg, Switzerland) with one end sealed and spun at 12 000 *g* for 5 min using a microhaematocrit centrifuge (Haematocrit 210 Centrifuge, Hettich, Bäch, Switzerland).

The haemoglobin concentration was assessed with the cyanmethaemoglobin method and measured using a photometer (Photometer LP 400, Dr Lange, Switzerland) after lysis of the red blood cells and centrifugation of the lysate for 5 min at 12 000 *g*, followed by colorimetric measurement at 540 nm. The MCHC was calculated from the PCV and the haemoglobin concentration.

Total WBC and red blood cell counts were obtained using a 1:200 blood dilution with Natt and Herrick solution and an improved Neubauer haemocytometer as previously reported (Carisch *et al.*, 2019).

### Clinical chemistry

The following biochemical parameters were measured in the plasma: total protein (TP), cholesterol, aspartate transaminase (AST), albumin (ALB), lactate dehydrogenase (LDH), uric acid and glucose. The tests were performed in 66 (TP, cholesterol, AST, LDH) and 39 (uric acid and glucose) animals, respectively (Supplemental Table 1).

All parameters were determined by colorimetry with an autoanalyser (Cobas Mira Roche autoanalyser, Hoffmann-La Roche Ltd, Basel, Switzerland), using commercially available kits (AST, cholesterol, LDH, TP, ALB: Diatools AG, Villmergen, Switzerland; uric acid: AxonLab, Dättwil, Switzerland). The glucose concentration was measured using a Roche Cobas c501 analyser (Roche Diagnostics, Schweiz AG, Rotkreuz, Switzerland).

### Corticosterone measurement

In 22 animals (Supplementary Table 1), plasma samples were analysed using a specific enzyme-immunoassay for corticosterone (Möstl *et al.*, 2002). A 150-μl aliquot of plasma was extracted with 3 ml of diethyl ether. After vortexing for 30 min, the plasma ether mixture was frozen at −20°C. The ether was poured into a new vial and evaporated to dryness, and then the residue was dissolved in 300-μl assay buffer. The extracted samples were analysed in duplicates. Serial dilutions of serum samples yielded parallel profiles with the standard curve.

### Statistical analysis

Haematological, plasma biochemical and corticosterone values were analysed using Stata 13 (StataCorp. 2013. *Stata Statistical Software: Release 13*. College Station, TX: StataCorp LP.). The level of significance testing was set with a *P* value of 0.05. Descriptive statistics were applied, and the data were tested for normality by the Shapiro–Wilk W test*.* For assessment of age-related differences, two age groups were established, young animals (<3 years of age; prior to definite sexual maturity and reproduction) and adult (≥3 years of age; sexually mature, reproducing animals).

Normally distributed data were analysed using *t*-test and ANOVA; non-parametric tests (Wicoxon rank-sum/Mann–Whitney) were used for data that were not normally distributed. Geometric and arithmetic mean, including confidence intervals, were determined for logarithmically transformed and raw data, respectively. Medians were reported where non-parametric tests are being used. Linear regression was used and linearity was confirmed using the qnorm plot and the Shapiro–Wilk W test on residuals.

Variables where data were normally distributed include the percentage of lymphocytes and azurophils, haematocrit, haemoglobin, MCHC, total protein, albumin, and glucose. Logarithmic transformation was applied on data for total number of leukocytes, number of lymphocytes, eosinophils, percentage of heterophils, eosinophils, monocytes and basophils, LDH, AST, urea, cholesterol and corticosterone. Nonparametric tests were used for number of azurophils, heterophils, monocytes and basophils and uric acid.

## Results

### Animals and disease/infection state

Thirty-one animals were young (<3 years of age; juvenile and subadult; Myers *et al.*, 2022); the remaining 48 animals were adult (≥3 years of age) (Table 1). Overall, the age of the animals ranged from 3 months to 20 years, with an average of 4.52 years. Both age groups comprised male and female animals in almost equal proportions; however, for 14 snakes, information on the sex either was not provided or could not be determined due to their young age (Supplementary Table 1).

**Table 1:** Leukocyte numbers in the peripheral blood (per μl) of clinically healthy captive *B. constrictor*.

**Cell type**	**Female**			**Male**			**Test para meters**	**Total**
	**<3 years**	**≥3 years**	**Total**	**<3 years**	**≥3 years**	**Total**		
	(*n*) Mean^*^ (95% CI)	(*n*) Mean (95% CI)	(*n*) Mean (95% CI)	(*n*) Mean (95% CI)	(*n*) Mean (95% CI)	(*n*) Mean (95% CI)		(*n*) Mean (95% CI)
**Leukocytes** ^(2)^ × 10^3^/μl	(3) **12.004** (7.009– 20.558)	(16) **9.561** (5.851– 15.624)	(20) **9.101** (5.945– 13.932)	(6) ** 7.418** (4.125– 13.338)	(11) **7.793** (5.210– 11.657)	(17) **7.659** (5.719– 10.257)	*t* = −0.6769, d*f* = 35, *P* = 0.5029	(49) **8.306** (6.768–10.193)
	*t* = 0.4162, d*f* = 17, *P* = 0.685			*t* = −0.1660, d*f* = 15, *P* = 0.8704				
**Lymphocytes ** ^(2)^ × 10^3^/μl	(3) ** 8.822** (3.142– 24.765)	(16) **3.877** (2.037– 7.381)	(20) **4.020** (2.295– 7.043)	(6) ** 3.640** (1.831– 7.238)	(11) **2.678** (1.680– 4.270)	(17) **2.984** (2.107– 4.227)	*t* = −0.9083, d*f* = 35, *P* = 0.3699	(49) **3.480** (2.679–4.522)
	*t* = 1.1424, d*f* = 17, *P* = 0.2691			*t* = 0.8873, d*f* = 15, *P* = 0.3889				
**Azurophils** ^(3)^ × 10^3^/μl	(3) ** 1.300** (0.150– 2.240) ^**^	(16) **2.245** (1.222– 4.996)	(20) **1.935** (0.637– 3.782)	(6) ** 1.275** (0.185– 5.756)	(11) **2.590** (1.344– 4.140)	(17) **2.340** (1.084– 3.319)	*z* = 0.411, *P* = 0.6807	(49) **1.880** (1.475–2.499)
	*z* = −1.230, *P* = 0.2188			*z* = −1.407, *P* = 0.1594				
**Heterophils** ^(3)^ × 10^3^/μl	(3) ** 1.250** (0.690– 1.460 ^**^	(16) **2.090** (0.963– 3.255)	(20) **1.900** (0.899– 2.776)	(6) ** 1.390** (0.290– 2-280)	(11) **2.290** (0.806– 3.777)	(17) **1.740** (0.830– 2.399)	*z* = −0.305, *P* = 0.7605	(49) **1.610** (1.040– 2.132)
	*z* = −1.342, *P* = 0.1797			*z* = −0.905, *P* = 0.3654				
**Eosinophils** ^(2)^ × 10^3^/μl	(3) ** 0.161** (0.013– 2.010)	(10) **0.144** (0.049– 0.421)	(14) **0.147** (0.069– 0.314)	(3) ** 0.129** (0.009– 1.804)	(7) ** 0.062** (0.032– 0.117)	(10) **0.077** (0.042– 0.140)	*t* = −1.3731, d*f* = 22, *P* = 0.1836	(34) **0.151** (0.098–0.231)
	*t* = 0.1208, d*f* = 11, *P* = 0.9060			*t* = 1.3394, d*f* = 8, *P* = 0.2172				
**Monocytes** ^(3)^ × 10^3^/μl	(3) **0.100** (0– 0.320) ^**^	(16) **0.180** (0.056– 0.260)	(20) **0.165** (0.043– 0.247)	(6) ** 0.360** (0.111– 1.582)	(11) **0.210** (0.121– 0.409)	(17) **0.220** (0.130–0.429)	*z* = 1.646, *P* = 0.0997	(49) **0. 210** (0.151–0.267)
	*z* = −0.615, *P* = 0.5384			*z* = 0.804, *P* = 0.4214				
**Basophils** ^(3)^ × 10^3^/μl	(3) **0** (0– 0.270) ^**^	(16) **0.220** (0.111– 0.324)	(20) **0.220** (0.095– 0.269)	(6) ** 0.065** (0.001– 0.287)	(11) **0.250** (0.093– 0.409)	(17) **0.220** (0.060– 0.260)	*z* = −0.259, *P* = 0.7953	(49) **0.230** (0.151–0.260)
	*z* = −1.345, *P* = 0.1788			*z* = −1.208, *P* = 0.2269				
**Cell type**	**<3 years**			**≥3 years**				**Total**
	**Female**	**Male**	**Total**	**Female**	**Male**	**Total**		
	(*n*) Mean^*^ (95% CI)	(*n*) Mean (95% CI)	(*n*) Mean (95% CI)	(*n*) Mean (95% CI)	(*n*) Mean (95% CI)	(*n*) Mean (95% CI)	**Test para meters**	(*n*) Mean (95% CI)
**Leukocytes** ^(2)^ × 10^3^/μl	(3) **12.004** (7.009–20.558)	(6) ** 7.418** (4.125–13.338)	(21) **8.296** (6.554–10.502)	(16) **9.561** (5.851–15.624)	(11) **7.793** (5.210–11.657)	(27) **8.797** (6.413–12.068)	*t* = −0.2915, d*f* = 46, *P* = 0.7719	(49) **8.306** (6.768–10.193)
	*t* = −1.3992, d*f* = 7, *P* = 0.2045			*t* = −0.6459, d*f* = 25, *P* = 0.5243				
**Lymphocytes ** ^(2)^ × 10^3^/μl	(3) ** 8.822** (3.142–24.765)	(6) ** 3.640** (1.831–7.238)	(21) **3.974** (2.916–5.414)	(16) **3.877** (2.037–7.381)	(11) **2.678** (1.680–4.270)	(27) **3.335** (2.218–5.013)	*t* = 0.6734, d*f* = 46, *P* = 0.5041	(49) **3.480** (2.679–4.522)
	*t* = −2.0990, d*f* = 7, *P* = 0.0740			*t* = −0.9137, d*f* = 25, *P* = 0. 3696				
**Azurophils** ^(3)^ × 10^3^/μl	(3) ** 1.300** (0.150–2.240)^**^	(6) ** 1.275** (0.185–5.756)	(21) **1.630** (1.035–2.122)	(16) **2.245** (1.222–4.996)	(11) **2.590** (1.344–4.140)	(27) **2.500** (1.859–3.425)	*z* = −1.673, *P* = 0.0943	(49) **1.880** (1.475–2.499)
	*z* = 0.258, *P* = 0.7963			*z* = 0.271, *P* = 0.7860				
**Heterophils** ^(3)^ × 10^3^/μl	(3) ** 1.250** (0.690–1.460^**^	(6) ** 1.390** (0.290–2-280)	(21) **1.270** (0.875–1.717)	(16) **2.090** (0.963–3.255)	(11) **2.290** (0.806–3.777)	(27) **2.230** (1.029–2.812)	*z* = −1.964, *P* < 0.05	(49) **1.610** (1.040–2.132)
	*z* = 0.519, *P* = 0.6041			*z* = −0.271, *P* = 0.7860				
**Eosinophils** ^(2)^ × 10^3^/μl	(3) **0.161** (0.013–2.010)	(3) ** 0.129** (0.009–1.804)	(16) **0.231** (0.130–0.410)	(10) **0.144** (0.049–0.421)	(7) ** 0.062** (0.032–0.117)	(17) **0.101** (0.053–0.196)	*t* = 1.9916, d*f* = 21, *P* = 0.0553	(34) **0.151** (0.098–0.231)
	*t* = −0.2606, d*f* = 4, *P* = 0.8072			*t* = −1.3863, d*f* = 45, *P* = 0.1859				
**Monocytes** ^(3)^ × 10^3^/μl	(3) ** 0.100** (0–0.320)^**^	(6) ** 0.360** (0.111–1.582)	(21) **0.230** (0.129–0.365)	(16) **0.180** (0.056–0.260)	(11) **0.210** (0.121–0.409)	(27) **0.190** (0.149–0.251)	*z* = 0.811, *P* = 0.4175	(49) **0. 210** (0.151–0.267)
	*z* = 1.549, *P* = 0.1213			*z* = 0.667, *P* = 0.5050				
**Basophils** ^(3)^ × 10^3^/μl	(3) ** 0** (0–0.270)^**^	(6) ** 0.065** (0.001–0.287)	(21) **0.220** (0.044–0.339)	(16) **0.220** (0.111–0.324)	(11) **0.250** (0.093–0.409)	(27) **0.230** (0.149–0.262)	*z* = −0.250, *P* = 0.8029	(49) **0.230** (0.151–0.260)
	*z* = 0.788, *P* = 0.4308			*z* = 0.099, *P* = 0.9213				

^*^The measures of position depend on data distribution: Arithmetic mean (1) for normally distributed data, geometric mean (2) for logarithmic transformation and median (3) for non-normal distribution.

^**^Lower (upper) confidence limit held at minimum (maximum) of sample.

**A)** Leukocyte concentrations in female and male *B. constrictor,* further divided according to the age groups (young: < 3 year; adult: ≥ 3 years). **B)** Leukocyte concentrations in young (< 3 years) and adult (≥ 3 years) *B. constrictor,* further divided by sex.

**Table 2:** Relative leukocyte percentages of clinically healthy captive *B. constrictor.*

**Cell type**	**Female**			**Male**				**Total**
	**<3 years**	**≥3 years**	**Total**	**<3 years**	**≥3 years**	**Total**		
	(*n*) Mean^*^ (95% CI)	(*n*) Mean (95% CI)	(*n*) Mean (95% CI)	(*n*) Mean (95% CI)	(*n*) Mean (95% CI)	(*n*) Mean (95% CI)	Test para meters	(*n*) Mean (95% CI)
**Lymphocytes** ^(1)^ (%)	(3) **74.5%** (35.70–100.00)	(16) **45.13%** (34.95–55.30)	(20) **49.20%** (39.44–58.96)	(6) **51.42%** (33.88–68.95)	(11) **35.73%** (28.34–43.12)	(17) **41.26%** (33.59–48.94)	*t* = −1.3089, df = 35, *P* = 0.1991	(49) **45.17%** (40.26–50.09)
	*t* = 2.4938, d*f* = 17, ***P* < 0.05**			*t* = 2.3456, d*f* = 15, ***P* < 0.05**				
**Azurophils** ^(1)^ (%)	(3) **11.50%** (13.99–36.99)	(16) **26.38%** (18.61–34.14)	(20) **22.98%** (15.87–30.08)	(6) **24.83%** (9.46–40.20)	(11) **31.27%** (26.48–36.06)	(17) **29.00%** (23.64–34.36)	*t* = 1.3813, d*f* = 35, *P* = 0.1759	(49) **25.95%** (22.21–29.69)
	*t* = −1.6721, d*f* = 17, *P* = 0.1128			*t* = −1.2361, d*f* = 15, *P* = 0.2354				
**Heterophils** ^(2)^ (%)	(3) **9.00%** (2.011–40.27)	(16) **18.32%** (13.17–25.47)	(20) **17.02%** **(**12.49–23.19)	(6) **14.28%** (7.11–28.68)	(11) **23.13%** (17.14–31.22)	(17) **19.51%** (14.60–26.09)	*t* = 0.6696, d*f* = 35, *P* = 0.5075	(49) **17.76%** **(**15.08–20.91)
	*t* = −1.8303, d*f* = 17, *P* = 0.0848			*t* = −1.7953, d*f* = 15, *P* = 0.0928				
**Eosinophils** ^(2)^ (%)	(3) **1.36%** (0.16–11.84)	(10) **1.46%** (0.88–2.43)	(14) **1.63%** (1.01–2.61)	(3) **1.82%** (0.46–7.22)	(7) **0.87%** (0.51–1.47)	(10) **1.08%** (0.69–1.72)	*t* = −1.2964, d*f* = 22, *P* = 0.2083	(34) **1.83%** (1.33–2.51)
	*t* = −0.1540, d*f* = 11, *P* = 0.8804			*t* = 1.8867, d*f* = 8, *P* = 0.0959				
**Monocytes** ^(2)^ (%)	(2) **1.73%** (0.002–100.00)	(15) **2.36%** (1.63–3.41)	(18) **2.22%** (1.61–3.07)	(6) **3.10%** (2.17–4.43)	(11) **2.76%** (1.76–4.30)	(17) **2.87%** (2.15–3.83)	*t* = 1.2452, d*f* = 33, *P* = 0.2218	(47) **2.50%** (2.08–3.00)
	*t* = −0.6110, d*f* = 15, *P* = 0.5503			*t* = 0.4027. d*f* = 15, *P* = 0.6929				
**Basophils** ^**(**2)^ (%)	(1) **2.5%**	(15) **2.27%** (1.46–3.52)	(17)** 2.54%** (1.63–3.94)	(10) **3.05%** (1.58–5.87)	(5) **1.47%** (0.38–5.72)	(15) **2.39%** (1.37–4.17)	*t* = −0.1818, d*f* = 30, *P* = 0.8569	(44) **2.61%** (1.96–3.47)
	N/A			*t* = −1.3723, d*f* = 13, *P* = 0.1932				
**Cell type**	**<3 years**			**≥3 years**				**Total**
	**Female**	**Male**	**Total**	**Female**	**Male**	**Total**		
	(*n*)Mean^*^ (95% CI)	(*n*) Mean (95% CI)	(*n*) Mean (95% CI)	(*n*) Mean (95% CI)	(*n*) Mean (95% CI)	(*n*) Mean (95% CI)	Test para meters	(*n*) Mean (95% CI)
**Lymphocytes** ^(1)^ (%)	(3) **74.5%** (35.70–100.00)	(6) **51.42%** (33.88–68.95)	(21) **50.48%** (42.75–58.20)	(16) **45.13%** (34.95–55.30)	(11) **35.73%** (28.34–43.12)	(27) **41.30%** (34.69–47.90)	*t* = 1.8759, d*f* = 46, *P* = 0.0670	(49) **45.17%** (40.26–50.09)
	*t* = −1.9900, d*f* = 7, *P* = 0.0869			*t* = −1.4678, d*f* = 25, *P* = 0.1546				
**Azurophils** ^(1)^ (%)	(3) **11.50%** (13.99–36.99)	(6) 24.83% (9.46–40.20)	(21) **23.93%** (17.89–29.96)	(16) **26.38%** (18.61–34.14)	(11) **31.27%** (26.48–36.06)	(27) 28.37% (23.56–33.19)	*t* = −1.2062 d*f* = 46, *P* = 0.2339	(49) **25.95%** (22.21–29.69)
	*t* = 1.3929, d*f* = 0.2063			*t* = 1.0284, d*f* = 25, *P* = 0.3136				
**Heterophils** ^(2)^ (%)	(3) **9.00%** (2.011–40.27)	(6) **14.28%** (7.11–28.68)	(21) **14.61%** (11.45–18.65)	(16) **18.32%** (13.17–25.47)	(11) **23.13%** (17.14–31.22)	(27) **20.14%** (16.15–25.12)	*t* = −2.0114 d*f* = 46, *P* = 0.0502	(49) **17.76%** **(**15.08–20.91)
	*t* = 1.0088, d*f* = 7, *P* = 0.3467			*t* = 1.0713, d*f* = 25, *P* = 032943				
**Eosinophils** ^(2)^ (%)	(3) **1.36%** (0.16–11.84)	(3) **1.82%** (0.46–7.22)	(16) **2.64%** (1.63–4.28)	(10) **1.46%** (0.88–2.43)	(7) **0.87%** (0.51–1.47)	(17) **1.18%** (0.83–1.68)	*t* = 2.8946, d*f* = 31, ***P* < 0.01**	(34) **1.83%** (1.33–2.51)
	*t* = 0.4889, d*f* = 4, *P* = 0.6505			*t* = −1.6073, d*f* = 15, *P* = 0.1288				
**Monocytes** ^(2)^ (%)	(2) **1.73%** (0.002–100.00)	(6) **3.10%** (2.17–4.43)	(20) **2.53%** (1.91–3.35)	(15) **2.36%** (1.63–3.41)	(11) **2.76%** (1.76–4.30)	(26) **2.52%** (1.93–3.28)	*t* = 0.0224, d*f* = 44, *P* = 0.9822	(47) **2.50%** (2.08–3.00)
	*t* = 1.6064, d*f* = 6, *P* = 0.1593			*t* = 0.5859, d*f* = 24, *P* = 0.5634				
**Basophils** ^(2)^ (%)	(1) **2.5%**	(10) **3.05%** (1.58–5.87)	(18) **2.45%** (1.46–4.12)	(15) **2.27%** (1.46–3.52)	(5) **1.47%** (0.38–5.72)	(25) **2.55%** (1.81–3.61)	*t* = −0.1454 d*f* = 41, *P* = 0.8851	(44) **2.61%** (1.96–3.47)
	N/A			*t* = 0.8581, d*f* = 23, *P* = 0.3997				

^*^The measures of position depend on data distribution: Arithmetic mean (1) for Normally distributed, Geometric mean (2) for logarithmic transformation and median (3) for non-normal distribution

**A)** Relative leukocyte percentages in female and male *B. constrictor,* further divided according to the age groups (young: < 3 year; adult: ≥ 3 years). **B)** Relative leukocyte percentages in young (< 3 years) and adult (≥ 3 years) *B. constrictor,* further divided by sex.

### Haematology

Haematological data were collected from 49 animals, 22 young and 27 adult boas. The differential WBC counts for the two age groups and for male and female snakes are provided in Tables 1–3.

**Table 3:** Blood parameters (haematocrit, haemoglobin, MCHC) of clinically healthy captive *B. constrictor*, divided by sex (upper table) and age (lower table)

	**Female**			**Male**				
**Para meter**	**<3 years**	**≥3 years**	**Total**	**<3 years**	**≥3 years**	**Total**	Test para meters	**Total**
	(*n*)Mean^*^ (95% CI)	(*n*) Mean (95% CI)	(*n*) Mean (95% CI)	(*n*) Mean (95% CI)	(*n*) Mean (95% CI)	(*n*) Mean (95% CI)		(*n*) Mean (95% CI)
**Haematocrit** ^(1)^	(3) **24.333** (10.208–38.459)	(14) **27.429** (25.864–28.993)	(18) **26.278** (24.204–28.351)	(6) **28.833** (26.313–31.353)	(8) **30.500** (26.478–34.522)	(14) **29.786** (27.519–32.052)	*t* = 2.4211, d*f* = 30, ***P* < 0.05**	(44) **26.023** (24.470–27.575)
	*t* = −1.4892, d*f* = 15, *P* = 0.1572			*t* = −0.7739, d*f* = 12, *P* = 0.4540				
**Haemoglobin ** ^(1)^	(3)** 7.033** (3.174–10.892)	(16) **8.101** (7.518–8.694)	(20) **7.805** (7.192–8.418)	(6) ** 8.417** (7.848–8.985)	(11) **9.473** (8.347–10.598)	(17) **9.100** (8.352–9.848)	*t* = 2.8486, d*f* = 35, ***P* < 0.01**	(49) **8.012** (7.546–8.479)
	*t* = −1.4631, d*f* = 17, *P* = 0.1617			*t* = −1.4830, d*f* = 17, *P* = 0.1588				
**MCHC ** ^(1)^	(3) **29.333** (27.899–30.768)	I16) **29.250** (27.593–30.907)	(20) **29.450** (28.096–30.804)	(6) **29.333** (27.166–31.501)	(11) **31.091** (28.605–33.577)	(17) **30.471** (28.794–32.148)	*t* = 1.0088, d*f* = 35, *P* = 0.3200	(49) **30.429** (29.567–31.290)
	*t* = 0.0452, d*f* = 17, *P* = 0.9644			*t* = −1.0662, d*f* = 15, *P* = 0.3032				
	**<3 years**			**≥3 years**				
	**Female**	**Male**	**Total**	**Female**	**Male**	**Total**		
	(*n*) Mean^*^ (95% CI)	(*n*) Mean (95% CI)	(*n*) Mean (95% CI)	(*n*) Mean (95% CI)	(*n*) Mean (95% CI)	(*n*) Mean (95% CI)		
**Haematocrit** ^(1)^	(3) **24.333** (10.208–38.459)	(6) **28.833** (26.313–31.353)	(21) **23.857** (21.635–26.079)	(14) **27.429** (25.864–28.993)	(8) **30.500** (26.478–34.522)	(22) **28.545** (26.854–30.236)	*t* = −3.5187 d*f* = 41, ***P* < 0.01**	(44) **26.023** (24.470–27.575)
	*t* = 1.7413, d*f* = 7, *P* = 0.1252			*t* = 1.9316, d*f* = 20, *P* = 0.677				
**Haemoglobin** ^ (1)^	(3)** 7.033** (3.174–10.892)	(6) ** 8.417** (7.848–8.985)	(21) **7.305** (6.671–7.938)	(16) **8.101** (7.518–8.694)	(11) **9.473** (8.347–10.598)	(27) **8.662** (8.070–9.257)	*t* = −3.2108 d*f* = 46, ***P* < 0.01**	(49) **8.012** (7.546–8.479)
	*t* = 2.0630, d*f* = 7, *P* = 0.780			*t* = 2.5628, d*f* = 25, ***P* < 0.05**				
**MCHC ** ^(1)^	(3) **29.333** (27.899–30.768)	(6) **29.333** (27.166–31.501)	(21) **30.857** (29.777–31.937)	(16) **29.250** (27.593–30.907)	(11) **31.091** (28.605–33.577)	(27) **30.000** (28.647–31.353)	*t* = 0.9789, d*f* = 46, *P* = 0.3327	(49) **30.429** (29.567–31.290)
	*t* = 0.0000, d*f* = 7, *P* = 1.000			*t* = 1.3997, d*f* = 25, *P* = 0.1739				

^*^The measures of position depend on data distribution: Arithmetic mean (1) for Normally distributed, Geometric mean (2) for logarithmic transformation and median (3) for non-normal distribution

**Figure 1:**
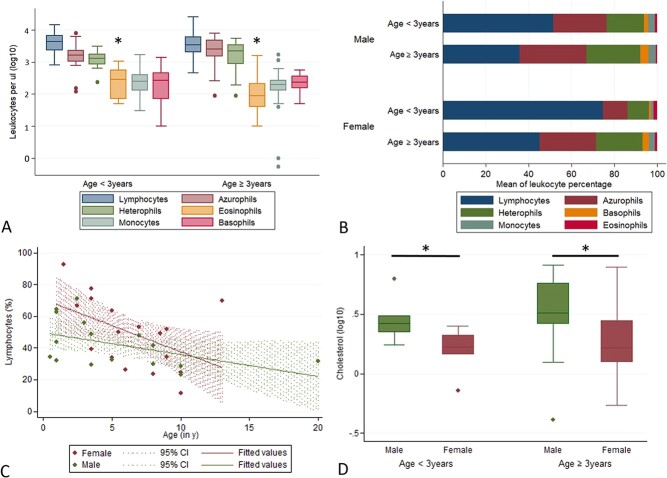
**Age- and sex-associated differences in blood parameters of healthy *B. constrictor***. **A, B. Distribution of leukocyte subpopulations. A.** Lymphocytes, azurophils and heterophils are the leukocyte subpopulations with the highest concentration in young (<3 years) and adult (≥3 years) *B. constrictor.* The concentration of eosinophils is significantly higher in the young animals. Box and whisker plots, ^*^P < 0.05. **B.** Lymphocytes, azurophils and heterophils are the dominant leukocyte subpopulations in both female and male *B. constrictor***.** Data provided as log-transformed values. **C. Age associated differences**. The percentage of lymphocytes shows a linear association and decreases with age. **D. Sex associated differences**. Male snakes show higher cholesterol levels than female snakes in both age groups. Box and whisker plots, ^*^P < 0.05.

Lymphocytes were the most abundant WBC in all animals, regardless of age and sex, followed by azurophils and heterophils; in contrast, the percentage of eosinophils, monocytes and basophils was always low and never exceeded 3% (Fig. 1 A, B). The younger animals (<3 years) showed a significantly lower percentage of eosinophils (geometric mean = 2.64%, CI = 1.63–4.28) than the adult animals (geometric mean = 1.18%, CI = 0.83–1.68, *t* = 2.895, d*f* = 31, *P* < 0.01) (Fig. 1A).

The percentage of lymphocytes showed a linear association with age and sex: *R*^2^ = 0.2421, *F*(2.31) = 4.95, *P* < 0.05 (lymphocyte percentage for female animals = 43.38 + 9.62 + (−2.006 × age), and for male animals = 43.38 + (−2.006 × age)) (Fig. 1C). The percentage of heterophils was significantly associated with the age group: *R*^2^ = 0.1809, F(2,33) = 3.64, *P* < 0.05, and higher in the adult boas.

Adult animals had a significantly higher haematocrit (mean = 28.56, CI = 26.85–30.24) and haemoglobin (mean = 8.66, CI = 8.01–9.26) than the young animals (mean = 23.86, CI = 21.64–26.08 with *t* = −3.5, d*f* = 41, *P* < 0.01 for haematocrit levels, and mean = 7.31, CI = 6.7–7.94 with *t* = 3.21, d*f* = 46, *P* < 0.01 for haemoglobin levels). Both parameters were significantly higher in males (mean = 29.79, CI = 27.52–30.05 for haematocrit, and mean = 9.1, CI = 8.35–9.85 for haemoglobin levels) than in females (mean = 26.28, CI = 24.2–28.35 with *t* = 2.42, d*f* = 30, *P* < 0.05 for haematocrit, and mean = 7.81, CI = 7.18–8.42 with *t* = 2.85, d*f* = 35, *P* < 0.01 for haemoglobin levels). Haemoglobin levels also showed a linear association with sex: *R*^2^ = 0.2064, *F*_2,31_ = 40.03, *P* < 0.05 (female: haemoglobin = 9.647–1.078 + 0.089 × age; male: haemoglobin = 9.647 + 0.089 × age).

All other haematological parameters did not vary significantly with age and sex (Tables 1–3).

### Identification and morphological features of blood cells

**Figure 2:**
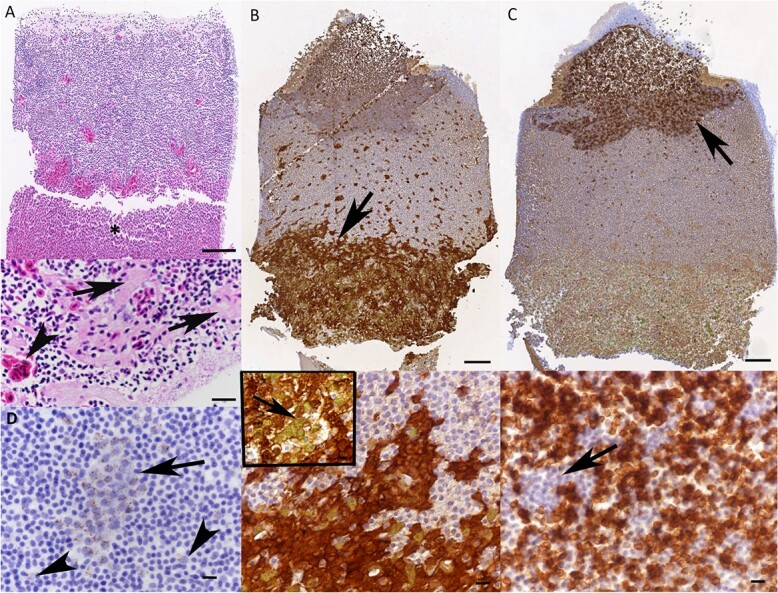
**Buffy coat of peripheral blood of healthy *B. constrictors* after formalin fixation and paraffin embedding. A.** HE-stained section of a buffy coat. Top: The blood cells do not arrange in distinct layers. Erythrocytes accumulate as a large aggregate below the buffy coat (asterisk). Bar = 100 μm. Bottom: The higher magnification shows the presence of small groups of erythrocytes (arrowhead) and small fibrinous aggregates (arrows) between the leukocytes. Bar = 25 μm. **B.** Iba-1, a confirmed marker of monocytes across animal classes, is expressed by numerous cells primarily at the bottom of the buffy coat (top, arrow). Due to their abundance, the Iba1-positive cells are interpreted as both monocytes and azurophils. Bottom: Strongly Iba1-positive monocytes/azurophils interspersed with numerous Iba1-negative heterophils, identified based on the presence of their distinct cytoplasmic granules (inset, arrow). Immunohistochemistry, haematoxylin counterstain. Bars = 100 μm (top) and 10 μm (bottom and inset). **C.** CD3, a confirmed marker of *T* cells across animal classes, is expressed by numerous cells primarily at the top of the buffy coat (top, arrow). Bottom: A higher magnification confirms the presence of abundant positive cells, interspersed with some aggregates of thrombocytes (arrow). Immunohistochemistry, haematoxylin counterstain. Bars = 100 μm (top) and 10 μm (bottom). **D.** RNA-ISH for CD20, a confirmed marker of B cells across animal classes, shows a signal in a low number of cells throughout the buffy coat, either as individual (arrowheads) or small groups (arrow) of round cells. RNA-ISH, haematoxylin counterstain. Bar = 10 μm.

**Figure 3:**
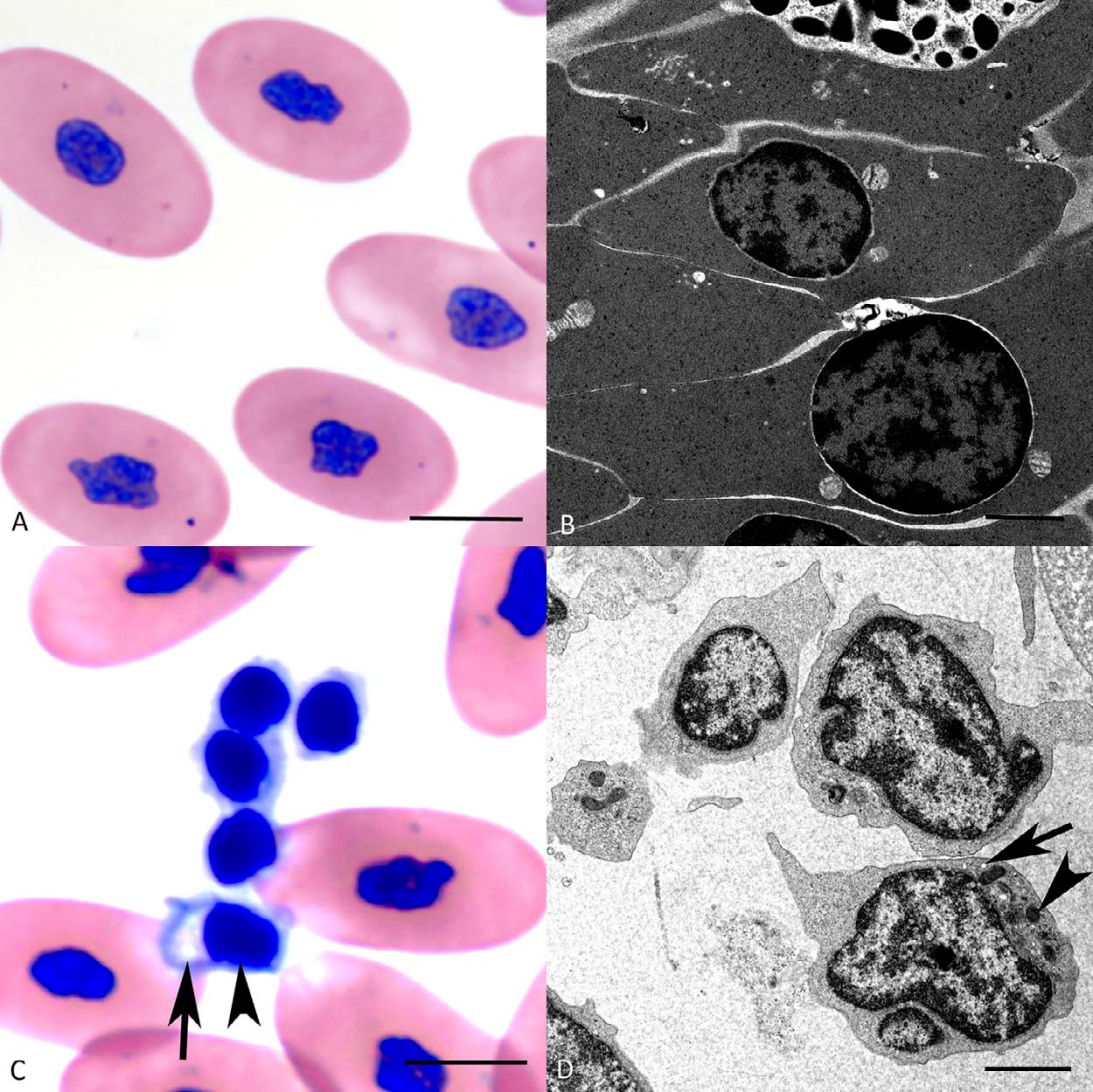
**Morphological features of erythrocytes and thrombocytes in the peripheral blood of healthy *B. constrictors*. A, B. Erythrocytes. A.** Cytological specimen. Erythrocytes are large, oval cells with a central oval nucleus and homogenous eosinophilic cytoplasm. **B.** TEM. Erythrocytes are elongated cells with uniformly electron-dense cytoplasm. The nucleus is round, with clumped heterochromatin that is arranged peripherally, along the nuclear membrane. **C, D. Thrombocytes. C.** Cytological specimen. Aggregate of four round thrombocytes, with small cytoplasmic pseudopodia and a round central nucleus (‘round’ form). There is also one thrombocyte with an ‘elongated’ form (arrowhead), a clear juxtanuclear cytoplasmic halo (arrow) and an oval nucleus. **D.** TEM of three thrombocytes. The cells are dominated by a central, irregularly outlined nucleus, a small amount of cytoplasm that contains small electron-dense vacuoles (arrowheads) and elongated to round more electron-lucent structures (‘canalicular structures’, arrow). A, C: May–Grünwald–Giemsa stain, bars = 10 μm; B, D: TEM, bars = 5 μm (C) and 2 μm (D).

**Figure 4:**
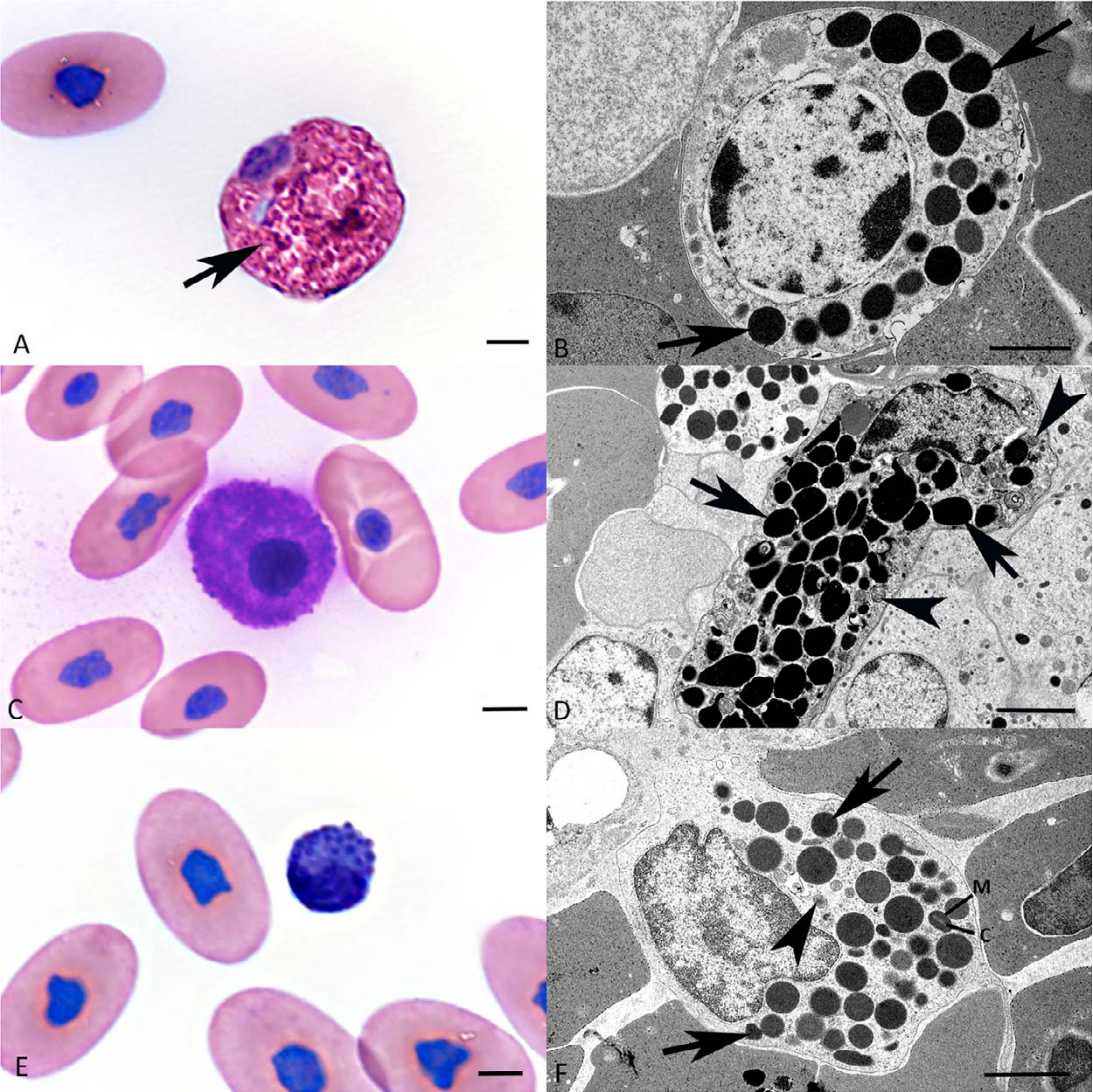
**Morphological features of heterophils, basophils and eosinophils in the peripheral blood of healthy *B. constrictor*. A, B. Heterophils. A.** Cytological specimen, showing a heterophil. The cytoplasm of the round cell is brightly eosinophilic and contains a moderate number of variably distinct granules (arrow); the nucleus is oval and eccentric. **B.** TEM shows abundant round electron dense cytoplasmic granules of variable size (arrows). The round nucleus is slightly peripherally located and shows marginalized clumps of heterochromatin. **C, D. Basophils.C.** Cytological specimen showing a basophil. The cell is slightly ovoid and exhibits abundant cytoplasm that is entirely filled by numerous round, distinct, intensely basophilic granules. **D.** Ultrastructurally, the granules are round and contain homogenous, highly electron-dense material (arrows). A few mitochondria (arrowheads) are also obvious. The nucleus is located peripherally and contains clumped heterochromatin. **E, F. Eosinophils.E. **Cytological specimen with an eosinophil. This round cell is smaller than heterophils and basophils, the cytoplasm is entirely filled by small indistinct darkly basophilic and eosinophilic granules. The nucleus is located in the periphery and appears slightly indented. **F.** TEM shows numerous round to elongated, variably sized granules in the cytoplasm filled with variably electron-dense material (arrows). Some granules show a central core (C) and a matrix (M). The nucleus has an irregular outline and abundant clumped heterochromatin. Arrowhead: mitochondrium. A, C, E: May–Grünwald–Giemsa stain, bars = 5 μm; B, D, F: TEM, bars = 2 μm.

**Figure 5:**
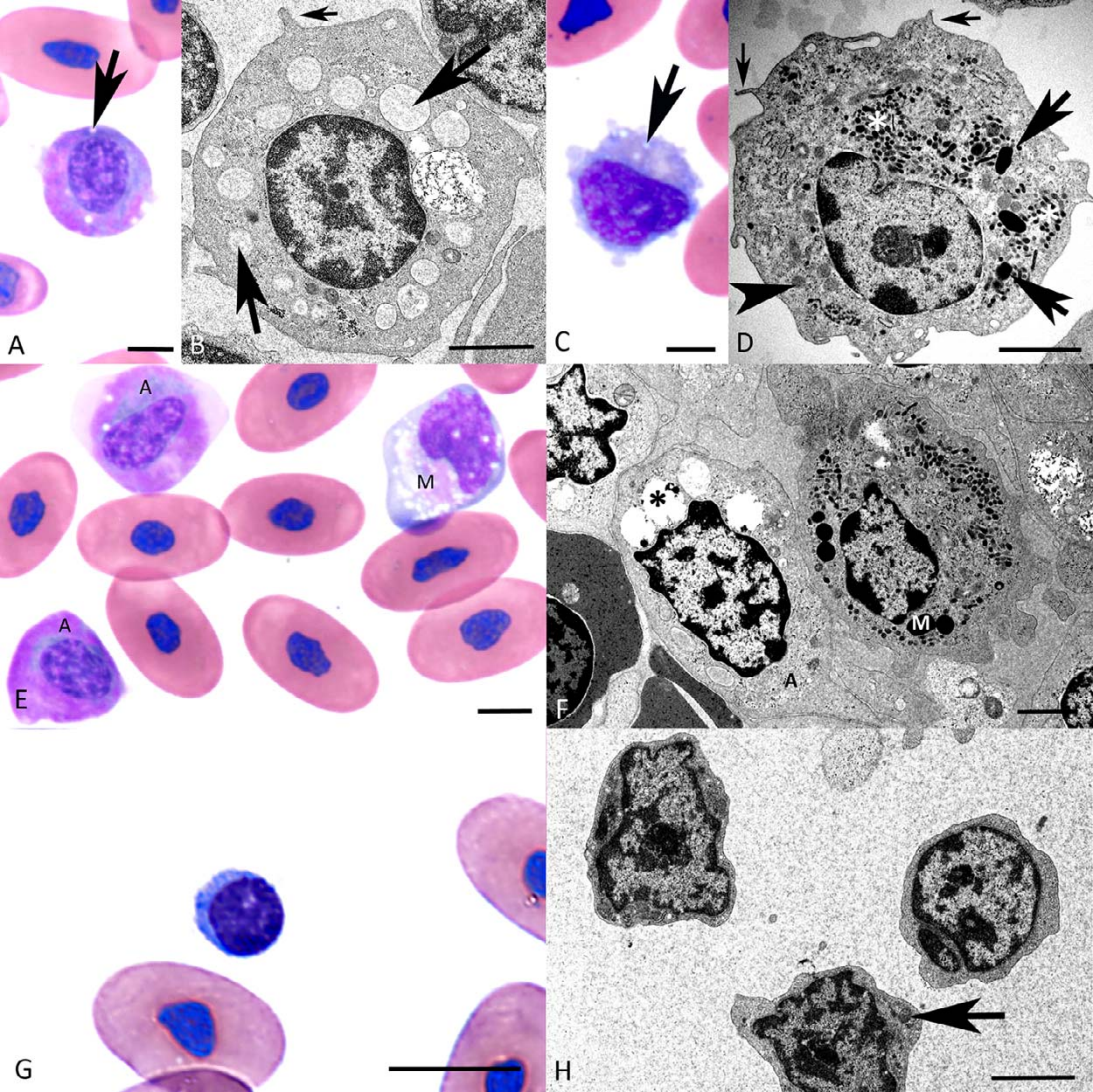
**Morphological features of azurophils and monocytes in the peripheral blood of healthy *B. constrictor*. A, B. Azurophils. A.** Cytological specimen showing an azurophil, a round cell with abundant variably basophilic to intensely eosinophilic cytoplasm that contains a few small clear vacuoles (arrow). The central nucleus is round, with clumped chromatin. **B.** Ultrastructure of an azurophil. The cell exhibits small cytoplasmic projections (small arrow) and variably sized cytoplasmic vacuoles that contain finely granular material of low electron density (arrow). **C, D. Monocytes. C.** Cytological specimen showing a monocyte. The round cell has abundant pale basophilic and moderately granular cytoplasm with a variable number of small vacuoles (arrow) and small cytoplasmic protrusions. The nucleus is eccentric and slightly indented. D. TEM image of a monocyte. There are several fine cytoplasmic protrusions (small arrows); the cytoplasm contains abundant organelles, amongst them small electron-dense round to elongated granules (asterisk), mitochondria (arrowhead), rough endoplasmic reticulum and lysosomes (arrows). The nucleus is eccentric and indented, with marginalized clumped chromatin and a distinct nucleolus. **E, F.** Direct comparison of azurophils and monocytes. **E. **In the cytological specimens, azurophils (A) present a darker, more homogenous, largely bright eosinophilic cytoplasm and a central, round nucleus. In contrast, the monocyte (M) has pale basophilic cytoplasm and an eccentric, indented nucleus. **F. **Ultrastructurally, the cytoplasm of the azurophil (A) is rich in vacuoles with variable content (asterisk), whereas the cytoplasm of the monocyte (M) is rich in organelles (mitochondria, rER, lysosomes) and contains abundant small electron dense granules. **G, H. Lymphocytes.G.** Cytological specimen with a lymphocyte, presenting as a small round cell with a round nucleus containing highly clumped chromatin; the cytoplasm is scant and basophilic. **H. **The cells exhibit scant cytoplasm with few mitochondria (arrow) and a round nucleus with large clumps of mainly peripheral heterochromatin. A, C, E, G: May–Grünwald–Giemsa stain, bars = 5 μm; B, D, F, H: TEM, bars = 2 μm.

The light microscopic examination of the buffy coats revealed no distinct layering of the leukocytes (Fig. 2), confirming a previous report (Fontes Pinto *et al.*, 2018). Apart from heterophils, which could be identified due to their prominent intracytoplasmic granules (Fig. 2B), differentiation of the leukocyte subpopulations based on their morphology was not possible, as cell borders, nuclear and/or cytoplasmic features could not be fully discerned (Fig. 2A). However, the immunohistochemical staining and RNA-ISH for *T* and B cells and monocytes (Fig. 2B-D), the cytological examination of the blood smears and the ultrastructural examinations of the buffy coat pellets allowed further identification of the different blood cell populations (Figs. 3-5).

#### Erythrocytes

The red blood cells formed a large aggregate below the buffy coat (Fig. 2A). Together with occasional variably sized fibrin clots, they were also present as small groups throughout all layers (Fig. 2A). Cytologically, erythrocytes presented as large oval uniform cells of consistent size (length: 12–16 μm, width: 5–7 μm), with a centrally located oval nucleus (Fig. 3A). Ultrastructurally, they were characterized by their elongate shape and uniformly electron-dense cytoplasm. Nuclei were round and uniform, with clumped heterochromatin that was arranged peripherally, along the nuclear membrane (Fig. 3B).

#### Thrombocytes

Thrombocytes were found on the top of the buffy coat. In the cytological specimens, they were 8–10 μm in diameter and varied in shape. Most were round with small cytoplasmic pseudopodia and a round central nucleus and often found in aggregates; others were elongate and exhibited a clear juxtanuclear cytoplasmic halo and an oval nucleus with dense chromatin (Fig. 3C). At ultrastructural level, thrombocytes were characterized by small electron-dense vacuoles and elongated to round electron loose structures (canalicular structures) in the cytoplasm (Israels SJ, 2007) and a round central nucleus (Fig. 3D).

**Heterophils** represented the most abundant granulocytes (Fig. 1A). They could be readily identified in the cytological specimens, as round cells with an eccentric round nucleus and a high number of sometimes refringent cytoplasmic granules with variable staining properties (Fig. 4A). Their size ranged between 15 and 25 μm in diameter. In the buffy coats, heterophils were mainly found in the bottom layer (Fig. 2B). TEM showed that the cytoplasm of heterophils is packed with granules; these are heterogeneous in shape, about 0.5–1 μm in diameter and of moderate to high electron density. Some heterophils also exhibited electron lucent vacuoles that contained irregularly shaped material (phagosomes/phagolysosomes) (data not shown). A few distinct organelles such as mitochondria were also observed. The nucleus was round, with marginalized clumps of heterochromatin (Fig. 4B).

**Basophils** were also readily identified in the cytological specimens, due to their abundant cytoplasm filled with numerous round, variably sized, intensely basophilic granules that occasionally masked the nucleus (Fig. 4C). They were 20–25 μm in diameter, round to oval, with a central to eccentric round nucleus. Ultrastructurally, the granules were 0.2–1 μm in diameter, round and of high electron density (Fig. 4D). Like in heterophils, the cytoplasm also contained a few distinct organelles such as mitochondria, and the nucleus was round, with marginalized clumps of heterochromatin.

**Eosinophils** were the smallest granulocytes (5–8 μm in diameter) in the cytological specimens (Fig. 4E). They were also round. The cytoplasm was more dense than in heterophils and contained abundant, variably distinct dark basophilic and eosinophilic granules. The nucleus was usually hyperchromatic and located in the periphery. TEM revealed an eccentric, ovoid nucleus with a moderate amount of peripheral heterochromatin. The abundant round to elongate cytoplasmic granules had a variably electron-dense homogenous content. Again, a few distinct organelles such as mitochondria were also identified in the cytoplasm (Fig. 4F).

#### Azurophils and monocytes

While monocytes in reptiles are generally thought to be morphologically and functionally similar to their mammalian counterpart, azurophils appear to be unique to reptile species (Vickie Joseph, 2015; Stacy *et al.*, 2011). Depending on the reptile order, however, they are described as morphologically (and possibly functionally) similar to both granulocytes and monocytes (Stacy *et al.*, 2011; Vickie Joseph, 2015). As previously described (Stacy *et al.*, 2011), azurophils and monocytes could be readily discerned in the cytological specimens (Fig. 5). **Azurophils** were round, 15–20 μm in diameter, with often brightly eosinophilic cytoplasm that contained occasional clear vacuoles. The nucleus was generally round and, different from basophils and eosinophils, centrally located, with clumped chromatin (Fig. 5A). **Monocytes** presented as roundish cells, with a slightly broader size range (12–25 μm in diameter). They exhibited small pseudopodia and a pale basophilic and moderately granular cytoplasm with or without vacuoles. The nucleus was central or eccentric and sometimes indented (Fig. 5C). Azurophils were by far more abundant than monocytes in the cytological specimens and could readily be differentiated from the latter due to the more eosinophilic cytoplasm (Fig. 5E).

In HE-stained buffy coat sections, however, monocytes and azurophils could not readily be discerned (Fig. 2A). Staining for Iba1, a marker of monocytes in a wide range of animal classes/species (Pierezan *et al.*, 2014) showed positive cells primarily in the bottom layer, suggesting these to be monocytes (Fig. 2B). However, considering the morphology of the Iba1-positive cells and their number and proportion in the haematological assessment, it appears likely that these represented both monocytes and azurophils (Fig. 2B). In contrast, heterophils, which could be readily discerned based on their distinct granules, were clearly Iba1 negative (Fig. 2B bottom, inset).

Also at ultrastructural level, monocytes and azurophils could readily be discerned (Fig. 5). The more abundant cells that were neither granulocytes nor lymphocytes, hence interpreted as azurophils, were 8–10 μm in diameter, with small cytoplasmic projections and vacuoles of variable number and size (0.5–2 μm) that contained finely granular material of low electron density (Fig. 5B and F). They occasionally contained cytoplasmic vacuoles filled with irregularly shaped material (phagosomes/phagolysosomes) and had a round nucleus with a significant amount of clumped heterochromatin. The second, less frequently encountered cells, hence interpreted as monocytes, were 8–12 μm in diameter and had an eccentric, often indented nucleus with a lesser amount of heterochromatin. Their cytoplasm formed thin projections and contained a high number of organelles, that is, mitochondria, rough endoplasmic reticulum and lysosomes, as well as abundant small electron-dense granules, and (Fig. 5D and F).

#### Lymphocytes

In the cytological specimens, lymphocytes presented as small- to medium-sized cells (5–10 μm in diameter) with a roundish outline and a central, round, purple to basophilic nucleus containing clumped chromatin (Fig. 5G). Immunohistochemical staining for CD 3 identified abundant *T* cells primarily at the top of the buffy coat (Fig. 2C). In contrast, B cells, identified based on the transcription of the B cell marker CD20 as shown by RNA-ISH, were rare and found individually or in small groups in the mid and upper layers of the buffy coat (Fig. 2D). This suggests that *T* cells are far more abundant than B cells in the peripheral blood of *B. constrictor*. The ultrastructural examination confirmed that lymphocytes have scant cytoplasm and few organelles, such as mitochondria, and a round nucleus with large clumps of mainly peripheral chromatin (Fig. 5F).

### Biochemical parameters and corticosteroid levels

Biochemical parameters (total protein, albumin, cholesterol, uric acid, glucose, urea, AST and LDH) were assessed in 66 animals, 27 young and 39 adult boas. The biochemical data for the two age groups and for male and female snakes (26 males and 25 females) are provided in Table 4.

Adult animals had significantly higher total protein (mean = 53.41, CI = 49.82–57) and albumin (mean = 30.37, CI = 28.55–31.19) levels than the young snakes (mean = 41.03, CI = 37.37–44.7 with *t* = −4.7, d*f* = 64, *P* < 0.0001 for total protein, and mean = 22.97, CI = 20.74–25.19 with *t* = −5.22, d*f* = 64, *P* < 0.0001 for albumin). The total protein level also showed an association with age (*R*^2^ = 0.1483, *F*_2,48_ = 4.18, *P* < 0.05), as older snakes showed higher levels than young snakes.

Male snakes generally showed higher cholesterol levels than female snakes regardless of age (young animals: males geometric mean = 2.82, CI = 1.96–4.07, females geometric mean = 1.56, CI = 0.858–2.85, with *t* = 2.33, d*f* = 10, *P* < 0.05; adult animals: males geometric mean = 3.12, CI = 2.21–4.14, females geometric mean = 1.94, CI = 1.4–2.66, with *t* = −2.13, d*f* = 37, *P* < 0.05); this is illustrated in Figure 1D. The association of cholesterol levels with sex was also confirmed (*R*^2^ = 0.1416, *F*_2,48_ = 3.96, *P* < 0.05).

**Table 4:** Biochemical parameters and corticosterone levels of clinically healthy captive *B. constrictor*.

	**Female**			**Male**				
**Para-meter**	**<3 years**	**≥3 years**	**Total**	**<3 years**	**≥3 years**	**Total**	Test para meters	**Total**
	(*n*) Mean^*^ (95% CI)	(*n*) Mean (95% CI)	(*n*) Mean (95% CI)	(*n*) Mean (95% CI)	(*n*) Mean (95% CI)	(*n*) Mean (95% CI)		(*n*) Mean (95% CI)
**Total protein** ^(1)^	(5) **38.680** (20.048–57.312)	(20) **53.030** (47.864–58.196)	(25) **50.160** (44.805–55.515)	(7) **44.843** (34.782–54.904)	(19) **53.468** (47.713–59.224)	(26) **51.146** (46.261–56.032)	*t* = 0.2809, d*f* = 49, *P* = 0.7800	(66) **48.533** (45.577–51.489)
	*t* = −2.4273, d*f* = 23, ***P* < 0.05**			*t* = −1.6696, d*f* = 24, *P* = 0.1080				
**Albumin** ^ (1)^	(5) **21.860** (12.622–31.098)	(20) **29.900** (26.936–32.864)	(25) **28.292** (25.323–31.261)	(7) **25.971** (19.591–32.352)	(19) **30.416** (28.077–32.755)	(26) **29.219** (26.919–31.519)	*t* = 0.5115, d*f* = 49, *P* = 0.6113	(66) **27.453** (25.815–29.091)
	*t* = −2.4591, d*f* = 23, ***P* < 0.05**			*t* = −1.8487, d*f* = 24, *P* = 0.0769				
**LDH** ^(2)^	(5) **68.367** (37.131–125.880)	(20) **112.309** (75.385–167.319)	(25) **101.696** (72.801–142.059)	(7) **115.381** (58.512–227.522)	(19) **100.631** (70.036–144.530)	(26) **104.406** (77.585–140.497)	*t* = 0.1215, d*f* = 49, *P* = 0.9038	(66) **105.845** (88.532–126.543)
	*t* = −1.2396, d*f* = 23, *P* = 0.2276			*t* = 0.4138, d*f* = 24, *P* = 0.6827				
**AST** ^(2)^	(5) **18.969** (4.994–72.056)	(20) **16.619** (11.041–25.016)	(25) **17.065** (11.797–24.685)	(7) **19.770** (12.644–30.912)	(19) **19.258** (14.241–26.042)	(26) **19.394** (15.333–24.532)	*t* = 0.6081, d*f* = 49, *P* = 0.5459	(66) **18.907** (15.821–22.597)
	*t* = 0.2900, d*f* = 23, *P* = 0.7744			*t* = 0.0999, d*f* = 24, *P* = 0.9212				
**Cholesterol** ^(2)^	(5) ** 1.564** (0.858–2.851)	(20) **1.935** (1.406–2.664)	(25) **1.855** (1.422–2.420)	(7) **2.822** (1.958–4.067)	(19) **3.122** (2.208–4.414)	(26) **3.038** (2.345–3.937)	*t* = 2.7396, d*f* = 49, ***P* < 0.01**	(66) **2.375** (2.030–2.779)
	*t* = −0.6534, d*f* = 23, *P* = 0.5200			*t* = −0.3500, d*f* = 24, *P* = 0.7294				
**Urea** ^(2)^	(5) ** 0.214** (0.111–0.411)	(18) **0.244** (0.174–0.343)	(24) **0.216** (0.159–0.293)	(7) **0.153** (0.076–0.309)	(19) **0.217** (0.149–0.315)	(25) **0.214** (0.159–0.289)	*t* = −0.0395, d*f* = 47, *P* = 0.9686	(61) **0.190** (0.155–0.232)
	*t* = −0.0344, d*f* = 22, *P* = 0.9729			*t* = −1.4983, d*f* = 23, *P* = 0.1477				
**Uric acid** ^(3)^	(5) ** 0.111** (0.068–0.369)^**^	(20) **0.244** (0.167–0.279)	(25) **0.242** (0.159–0.274)	(7) ** 0.212** (0.087–0.446)	(19) **0.180** (0.130–0.297)	(26) **0.189** (0.137–0.296)	*z* = −0.104, *P* = 0.9175	(66) **0.200** (0.167–0.275)
	*z* = −1.223, *P* = 0.2214		*z* = 0.434, *P* = 0.6646					
**Glucose** ^ (1)^	(2) ** 3.100** (0–17.077)	(10) **1.940** (1.202–2.678)	(13) **2.062** (1.385–2.738)	(5) ** 3.300** (2.016–4.584)	(9) ** 2.044** (1.311–2.778)	(14) **2.493** (1.840–3.146)	*t* = 0.9948, d*f* = 25, *P* = 0.3294	(39) **2.3** (1.947–2.653)
	*t* = 1.3668, d*f* = 10, *P* = 0.2016			*t* = 2.293, d*f* = 12, ***P* < 0.05**				
**Corticosterone** ^ (2)^	(7) ** 3.52** (1.881–6.594)	(7) ** 2.980** (1.259–7.058)	(11) **3.246** (1.833–5.749)	(3) **2.427** (0.639–9.223)	(4) ** 3.769** (1.068–13.304)	(10) **3.150** (2.000–4.960)	*t* = −0.0913, d*f* = 19, *P* = 0.9282	(22) **3.246** (2.356–4.471)
	*t* = 0.4219, d*f* = 9, *P* = 0.6830			*t* = −0.8349, d*f* = 8, *P* = 0.4280				
**Para meter**	**<3 years**			**≥3 years**				**Total**
	**Female**	**Male**	**Total**	**Female**	**Male**	**Total**	Test para meters	
	(*n*) Mean^*^ (95% CI)	(*n*) Mean (95% CI)	(*n*) Mean (95% CI)	(*n*) Mean (95% CI)	(*n*) Mean (95% CI)	(*n*) Mean (95% CI)		(*n*) Mean (95% CI)
**Total protein ** ^(1)^	(5) **38.680** (20.048–57.312)	(7) **44.843** (34.782–54.904)	(26) **41.031** (37.367–44.695)	(20) **53.030** (47.864–58.196)	(19) **53.468** (47.713–59.224)	(40) **53.410** (49.815–57.005)	*t* = −4.7042, d*f* = 64, ***P* < 0.0001**	(66) **48.533** (45.577–51.489)
	*t* = 0.8293, d*f* = 10, *P* = 0.4263			*t* = 0.1191, d*f* = 37, *P* = 0.9058				
**Albumin** ^(1)^	(5) **21.860** (12.622–31.098)	(7) **25.971** (19.591–32.352)	(26) **22.969** (20.740–25.199)	(20) **29.900** (26.936–32.864)	(19) **30.416** (28.077–32.755)	(40) **30.368** (28.549–32.186)	*t* = −5.2232, d*f* = 64, ***P* < 0.0001**	(66) **27.453** (25.815–29.091)
	*t* = 0.9862, d*f* = 10, *P* = 0.3473			*t* = 0.2844, d*f* = 37, *P* = 0.7777				
**LDH** ^(2)^	(5) **68.367** (37.131–125.880)	(7) **115.381** (58.512–227.522)	(26) **102.920** (79.762–132.800)	(20) **112.309** (75.385–167.319)	(19) **100.631** (70.036–144.530)	(40) **107.791** (83.733–138.761)	*t* = −0.2508, d*f* = 64, *P* = 0.828	(66) **105.845** (88.532–126.543)
	*t* = 1.3790, d*f* = 10, *P* = 0.1980			*t* = −0.4259, d*f* = 37, *P* = 0.6727				
**AST** ^(2)^	(5) **18.969** (4.994–72.056)	(7) **19.770** (12.644–30.912)	(26) **20.016** (15.197–26.363)	(20) **16.619** (11.041–25.016)	(19) **19.258** (14.241–26.042)	(40) **18.220** (14.298–23.218)	*t* = 0.5118, d*f* = 64, *P* = 0.6105	(66) **18.907** (15.821–22.597)
	*t* = 0.0910, d*f* = 10, *P* = 0.9293			*t* = 0.6025, d*f* = 37, *P* = 0.5505				
**Cholesterol** ^(2)^	(5) ** 1.564** (0.858–2.851)	(7) ** 2.822** (1.958–4.067)	(26) **2.205** (1.832–2.654)	(20) **1.935** (1.406–2.664)	(19) **3.122** (2.208–4.414)	(40) **2.493** (1.971–3.153)	*t* = −0.7600, d*f* = 64, *P* = 0.4500	(66) **2.375** (2.030–2.779)
	*t* = 2.3296, d*f* = 10, ***P* < 0.05**			*t* = 2.1302, d*f* = 37, ***P* < 0.05**				
**Urea** ^(2)^	(5) **0.214** (0.111–0.411)	(7) **0.153** (0.076–0.309)	(23) **0.148** (0.106–0.207)	(18) **0.244** (0.174–0.343)	(19) **0.217** (0.149–0.315)	(38) **0.221** (0.172–0.283)	*t* = −1.9731, d*f* = 59, *P* = 0.0532	(61) **0.190** (0.155–0.232)
	*t* = −0.8491, d*f* = 10, *P* = 0.4157			*t* = 0.5009, d*f* = 35, *P* = 0.6196				
**Uric acid** ^(3)^	(5) ** 0.111** (0.068–0.369)**	(7) ** 0.212** (0.087–0.446)	(26) **0.239** (0.121–0.349)	(20) **0.244** (0.167–0.279)	(19) **0.180** (0.130–0.297)	(40) **0.208** (0.160–0.275)	*z* = 0.623, *P* = 0.5330	(66) **0.220** (0.167–0.275)
	*z* = 0.731, *P* = 0.4649			*z* = −0.604, *P* = 0.5457				
**Glucose** ^ (1)^	(2) ** 3.100** (0–17.077)	(5) **3.300** (2.016–4.584)	(19) **2.668** (2.128–3.209)	(10) **1.940** (1.202–2.678)	(9) ** 2.044** (1.311–2.778)	(19) **1.989** (1.522–2.457)	*t* = 1.9973, d*f* = 36, *P* = 0.0534	(39) ** 2.3** (1.947–2.653)
	*t* = 0.2065, d*f* = 5, *P* = 0.8445			*t* = 0.2282, d*f* = 17, *P* = 0.8222				
**Corticosterone** ^ (2)^	(7) **3.52** (1.881–6.594)	(3) ** 2.427** (0.639–9.223)	(7) ** 3.121** (1.661–5.867)	(7) ** 2.980** (1.259–7.058)	(4) ** 3.769** (1.068–13.304)	(15) **3.306** (2.166–5.044)	*t* = −0.1692, d*f* = 20, *P* = 0.8673	(22) **3.246** (2.356–4.471)
	*t* = −0.8211, d*f* = 5, *P* = 0.4489			*t* = 0.3830, d*f* = 12, *P* = 0.7084				

^*^The measures of position depend on data distribution: Arithmetic mean (1) for Normally distributed, Geometric mean (2) for logarithmic transformation and median (3) for non normal distribution

^**^ Lower (upper) confidence limit held at minimum (maximum) of sample

**(A)** Biochemical parameters and corticosterone levels for young (< 3 years) and adult (≥ 3 years) *B. constrictor,* further divided by sex. (**B)** Biochemical parameters and corticosterone levels for young (< 3 years) and adult (≥ 3 years) *B.constrictor,* further divided by sex.

All other biochemical parameters as well as the corticosterone levels did not vary significantly with age and sex (Table 4).

## Discussion

This study aimed to establish reference data for the peripheral blood of captive *B. constrictor.* A wide range of biochemical and haematologic variables, plasma corticosterone levels as well as the morphology of the peripheral blood cells were investigated. An age and sex group distinction was applied due to the commonly known fact that regardless of animal classes or species, total WBC concentration can differ significantly between young and adult animals and between sexes, the former possibly being related to sexual maturity (Wood, 2014; Raskin *et al.*, 2004). Multiple studies in mammals, birds and fewer in reptiles have highlighted the importance of age-related reference values for the evaluation of leukograms (Lee *et al.*, 2020; Fonseca Sarmiento *et al.*, 2018; Trimboli *et al.*, 2020; Dolka *et al.*, 2014). We chose 3 years as the cut-off, because captive *B. constrictor* are known to reach sexual maturity around 3 years of age. This does not necessarily apply to their wild counterparts, as here, the onset of reproductive activity is strongly dependent on the season (Bertona and Chiaraviglio, 2003).

In the wild, *B. constrictor* can be found in the tropical forests of Central and South America on altitudes between 0 and 1500 metres above sea level, where there are strong seasonal changes in precipitation intensity (dry season: March to November; wet season: December to February) and an ambient temperature that does not fluctuate substantially. Hence, previous studies on the haematological and biochemical parameters of wild reptiles comprized two sampling time points (winter/summer season) (el Masri *et al.*, 1995; Holding *et al.*, 2014; Hussein *et al.*, 1979; Leceta *et al.*, 1989; Leceta and Zapata, 1985; Silva *et al.*, 2011; Machado *et al.*, 2006). Such an approach was not taken in our study, which included samples collected at ad hoc time points during the year, as to our knowledge the housing conditions of captive snakes in Switzerland and Germany do not mimic seasonal variations. While maintenance guidelines for *B. constrictor* are closely adapted to the conditions in the wild and often recommend 50–80% humidity and an air temperature of 24–32°C (Hedley, 2022), personal communication with breeders indicates that *B. constrictor* is commonly held at the same temperature throughout the year, with slight individual temperature differences between day and night.

The haematological examination in the present study yielded leukocyte subset concentrations that overall aligned with those reported for captive and wild boid snakes so far (Machado *et al.*, 2006; Fonseca Sarmiento *et al.*, 2018; Jenkins-Perez, 2012). In comparison to previous reports on captive and wild boas, our total blood leukocyte numbers were relatively high (in the upper third of the values published so far). However, the WBC numbers were similar to those shown in the few other studies on captive *B. constrictor* in which this ‘leukocytosis’ was speculated to be related to the stress induced by captivity (Machado *et al.*, 2006; Brenner *et al.*, 2002). Interestingly, a study on wild boids (*Corallus hortulanus)* indeed reported notably lower WBC counts (in the lower third of the values published so far) (Quadrini *et al.*, 2018).

In the most common domestic mammal species, neutrophils (dogs, cats, horses etc.) or lymphocytes (pigs, cows) are the dominating leukocyte subpopulations in the peripheral blood of healthy individuals, with monocytes, eosinophils and basophils comprising a noticeably smaller proportion (Wood, 2014). In reptiles, lymphocytes (comprising up to 80% of all leukocytes), heterophils and azurophils, the latter representing a cell type unique to reptiles (Stacy *et al.*, 2011), have been described as the most frequent leukocyte subpopulations. This was confirmed by the present study that identified lymphocytes as the most abundant leukocyte subtype regardless of age and sex. We also observed a correlation of the lymphocyte proportion with age, as younger snakes presented a higher percentage of circulating lymphocytes. This is not surprising considering that young mammals (ruminants, dogs etc.) normally have higher lymphocyte concentrations than older animals (Wood, 2014). As it does not represent a pathologic process, this physiologic variation is often termed ‘*pseudolymphocytosis’* of young animals (Boes *et al.*, 2017). In humans, the establishment of the adaptive immune system is initiated after birth, and the juvenile haematopoiesis becomes lymphoid biassed, which correlates with an increase in the total number of *T* lymphocytes by thymic expansion (Holcar *et al.*, 2015). The results of the present study, as well as those of previous studies on other reptile species (Fonseca Sarmiento MVZ *et al.*, 2018; Page-Karjian *et al.*, 2021; Casal *et al.*, 2009), indicate a similar phenomenon also in the class reptilia. Interestingly, the adult boas of the present study showed a higher lymphocyte percentage than a cohort of captive amazon tree boas previously studies (*Corallus hortulanus*) (Quadrini *et al.*, 2018). Whether this reflects real differences between closely related species remains unclear but would be suggested, because all examined animals were clinically healthy, without evidence of inflammatory processes, infectious disease or ecdysis, which rules out most of the reported causes of lymphocytosis in reptiles (Stacy *et al.*, 2011; Vickie Joseph, 2015).

In contrast to the lymphocyte percentages, we observed an increase in the percentage of heterophils with age. This finding is in accordance with a study performed on Brazilian wild *B. constrictor* (Fonseca Sarmiento *et al.*, 2018). In humans, haematopoiesis becomes myeloid biassed with increasing age and sexual maturation, an effect that is regulated by the influence of extrinsic and intrinsic factors on the haematopoietic stem cells and multipotent progenitor cells (Wang *et al.*, 2022; Ho *et al.*, 2019; Heo *et al.*, 2015).

We observed significantly higher haematocrit and haemoglobin levels in adult and in male snakes. In mammals, the haematocrit is also known to increase with age/maturity (Cornell *et al.*, 2017; Eklom and Lill, 2006), a fact that has been attributed to developmental changes in the haematopoietic system (Sealander, 1965). Studies on humans and other mammals have also reported higher haemoglobin levels in male individuals; this is thought to be due to the stimulatory effect of androgens on the bone marrow (Wahed and Dasgupta, 2015). Interestingly, lower haematocrit and haemoglobin values were reported in adult compared to juvenile wild *B. constrictor* from Colombia (Fonseca Sarmiento *et al.*, 2018); yet, unfortunately, information on exact age and age group definition was not provided for these animals. There is an overall paucity of data on the age-related changes of haematological variables in reptiles in general and a direct comparison with mammals might be inappropriate, due to differences in evolutionary development. As an example, the overall life span of reptile erythrocytes has been stated to be 600–800 days, depending on the species (Vickie Joseph, 2015), whereas it is around 20–160 days in mammals (Vácha and Znojil, 1981). Therefore, more studies, with a variety of reptile species and larger any cohorts of different age groups are needed to consolidate the findings of this and previous studies.

Overall, the biochemical values obtained from the present boa constrictor cohort were in line with those formerly reported for this species (Chiodini and Sundberg, 1982). Also, the significantly higher total protein levels in the older snakes are consistent with the relative total protein increase with age and weight that has been reported in turtles, tortoises and iguanas (Casal *et al.*, 2009; Harr *et al.*, 2001; Dickinson *et al.*, 2002). We observed variable cholesterol levels observed in our group of snakes. Similar to observations in Nile crocodiles (*Crocodylus niloticus)* and loggerhead sea turtles *(Caretta caretta)*, we found the these to be higher with age and in males (Rousselet *et al.*, 2013; Morpurgo and Gelman, 1991). In humans, decreased low density lipoprotein (LDL)-receptor activity is responsible for the increase in total cholesterol and LDL levels observed with age, whereas the generally lower levels in women are mostly due to the influence of oestrogen (Downer *et al.*, 2014).

Circulating corticosterone levels are widely accepted as biomarkers for stress in vertebrates (Claunch *et al.*, 2017), and plasma corticosterone levels are commonly used to quantify acute stress in snakes (Gangloff *et al.*, 2017; Neuman-Lee *et al.*, 2020); however, an elevation in baseline blood corticosterone has also been stated to be an indicator of chronic stress in snakes (van Waeyenberge *et al.*, 2018). There is also the assumption that chronically stressed animals will show a greater increase in stress hormones when subjected to acute stimuli, such as blood sampling (Dantzer *et al.*, 2014). The potential elevation of plasma corticosterone levels due to capture and handling (Herr *et al.*, 2017) brings in a bias that needs to be considered also in our study, since handling of the snakes for blood sampling was within a time frame of ~5 min (never exceeding 10 min). Interestingly though, the corticosterone levels determined in our cohort were overall much lower than those reported in wild, free-ranging snakes, such as rattlesnakes, dice snakes and watersnakes at the time of blood sampling (Lakušić *et al.*, 2020; Claunch *et al.*, 2017; McCallie and Klukowski, 2022; Lind *et al.*, 2018). This would indicate that captive snakes, which are at least used to handling, are not significantly stressed by the blood sampling. However, the interpretation of stress hormone levels is challenging, as they are influenced by a range of factors, such as age and nutritional status, all well described in mammals (Reeder and Kramer, 2005), but can also be affected by numerous other, less described factors in birds and reptiles, including the type of environment, environmental temperature and humidity, handling and the frequency of feeding (Jaffredo *et al.*, 2006; Holding *et al.*, 2014). Due to these limitations and the lack of similar studies in wild boa constrictors, it is difficult to comment to which extent the more ‘standardized’ housing conditions and the frequent handling by/habituation to humans of the captive snakes in this study may indeed have contributed to the observed low overall corticosterone and therefore likely low stress levels.

We used fixed and processed buffy coat preparations to gather detailed information on leukocyte subsets in boa constrictors, following a previously reported approach to the examination of blood cells in several reptile species (Fontes Pinto *et al.*, 2018). Our results confirm that a distinction of leukocyte subpopulations is not possible on HE stained sections prepared after paraffin embedding, confirming that cytological specimens obtained from blood smears are most suited for this endeavour. However, using immunohistochemistry and RNA-ISH on the buffy coat preparations, we were able to identify *T* and B cells for the first time. The results show that *T* cells are markedly more numerous than B cells in the peripheral blood of healthy boas. Since there are so far no studies on lymphocyte subpopulations in reptiles, we rely on the finding in other animal classes to put this result into context. Indeed, also in birds, i.e. in chickens, the order phylogenetically closest to reptiles, *T* cells are far more numerous (> 20%) in the peripheral blood than B cells (maximally 10%) (Hao *et al.*, 2020). Expression of the *T* cell marker CD3 was observed in approximately 12–24% (average) of the lymphoid cells in the blood of chickens, with a dominance of CD4^+^*T* helper cells over CD8^+^ cytotoxic *T* cells (Fair *et al.*, 2008). In the present study, we decided against an attempt to further determine lymphocyte subsets due to the lack of antibodies for reptiles. More detailed examinations on the haemolymphatic organs of *B.constrictor* are now needed to determine the general distribution of lymphocyte subtypes.

We used a rabbit polyclonal antibody against human Iba1, a calcium binding protein that is upregulated during the activation of macrophages, that we have previously shown to cross react with snakes (Dervas *et al.*, 2020), to detect monocytes in the boas. Interestingly, the marker appeared to be expressed also by azurophils (indirect evidence from their frequency and morphology in the blood smear), which, along with heterophils, thrombocytes and B cells, are considered as phagocytic cells in reptiles (Vickie Joseph, 2015, Zimmerman *et al.*, 2010). The literature on the classification of monocytes and azurophils in snakes is contradictory. Some authors state that azurophils should be counted separately in snakes, as their staining properties (e.g. they are positive in both the PAS and acid phosphatase reaction) render them more similar to mammalian neutrophils (Zimmerman et al., 2010; Campbell and Grant, 2022). However, because their granulopoietic origin has not been confirmed, others claim that monocytes and azurophils should not be looked upon separately, as the azurophils might represent immature monocytes (Vickie Joseph, 2015). Similar to other studies, we also found azurophils to be by far more abundant in the differential blood count (second most common leukocyte subtype) than monocytes (Nardini *et al.*, 2013; Stacy *et al.*, 2011). It was possible to discern them from monocytes in the peripheral blood smear as they showed a darker basophilic cytoplasm. The very limited literature on the ultrastructural features of monocytes and azurophils (Martinez Silvestre *et al.*, 2005; Salakij *et al.*, 2002), however, did so far not allow direct conclusions on the appearance of the two cell types in *Boa constrictor.* The present study sheds light on these, identifying monocytes as cells with clear cytoplasmic granules, similar to those described in human bone marrow promonocytes, blood monocytes and macrophages (Collin *et al.*, 2016). Azurophils, in contrast, showed intracytoplasmic vacuoles and/or phagocytosed material (indicating phagocytic activity), but lacked intracytoplasmic granules. Taken together, our findings suggest that azurophils do not represent an earlier/different developmental stage of a monocyte but constitute a distinct, fully differentiated, phagocytically active blood cell type in *B. constrictor* that might indeed originate from the monocytic lineage. Further studies are required to fully determine the origin and function of azurophils vs monocytes in boas and in snakes in general, and to determine whether they are still apparent in inflammatory processes.

In summary, the present study provides reference values for relevant haematological and biochemical markers as well as stress hormone levels in healthy captive *B. constrictor,* taking into account age- and sex-related differences. Because data were obtained under ‘standardized’ captive conditions, they should be useful for studies on diseased captive boas and to compare captive and wild boa populations. The in-depth morphological approach to characterize the cells of the peripheral blood, using a combined cytologic, histologic, immunohistochemical, RNA-ISH and ultrastructural approach can pave the way for further research on leukocyte functions, the composition of haemolymphatic tissues in boa constrictors which have, despite its large captive global population and distribution, so far not been elucidated. They can also serve towards studies on immunopathological processes in this species.

## Data availability statement

Datasets are available on request**:** The raw data supporting the conclusions of this article will be made available by the authors, without undue reservation.

## Supplementary Material

Web_Material_coad001
