# Supplementary material for: Haematology, biochemistry and morphological features of peripheral blood cells in captive Boa constrictor
Source: Conserv Physiol. 2023 Jan 28;11(1):coad001. doi: 10.1093/conphys/coad001 (PMC9885740; doi:10.1093/conphys/coad001)
Supplement: Web_Material_coad001 [file web_material_coad001.zip › Supplemental_Table_4.docx]

**Supplemental Table S4**. List of animals (clinically healthy *B. constrictor*) examined as part of the study. The capital letters in front of the animal numbers represent the different breeding colonies or private collections (A-L): Swiss breeding colonies/collections, M: German breeding colony). Peripheral blood samples from the animals were subjected to the following investigations: hematology (H), biochemical analysis (B), determination of glucose (G) and corticosterone (C) levels. In selected cases, buffy coats were prepared and subjected to morphological examinations (hematoxylin eosin stained section, immunohistology to determine T cells and monocytes, RNA-ISH to detect B cells, (BC)) and ultrastructural examination (U).

| **Animal No** | **Age** | **Sex** | **Length (m)** | **Weight (g)** | **Investigations** |
| --- | --- | --- | --- | --- | --- |
| A1 | 1 y | nd | 0.67 | 108 | H, BC |
| A2 | 1 y | nd | 0.7 | 114 | H, B, G, BC |
| A3 | 1 y | nd | 0.6 | 91 | H, B, G, BC |
| A4 | 1 y | nd | 0.65 | 123 | H, B, G, BC |
| A5 | 1 y | nd | 0.82 | 183 | H, B, G, BC |
| A6 | 1 y | nd | 0.8 | 191 | H, B, G |
| A7 | 1 y | 1.0 | 0.82 | 226 | H, B, G |
| A8 | 1 y | 1.0 | 0.72 | 131 | H, B, G |
| A9 | 1 y | 1.0 | 0.69 | 117 | H, B, G |
| A10 | 1 y | 0.1 | 1.1 | 132 | H, B, G |
| A12 | 3 y | 0.1 | 1.2 | 2,500 | H, B, G |
| A14 | 3.5 y | 0.1 | 1.4 | 2,500 | H, B, G |
| A15 | 3.5 y | 1.0 | 1.3 | 1,500 | B, C |
| A16 | 3.5 y | 1.0 | 1.3 | 1,500 | B, C |
| A17 | 3.5 y | 1.0 | 1.3 | 1,500 | B, C |
| A18 | 5 y | 0.1 | 1.7 | 5,000 | B, C |
| A19 | 13 y | 0.1 | 2 | 4,000 | H, B, G |
| A20 | 7 y | 0.1 | 2 | 4,000 | H, B, G |
| A21 | 3.5 y | 1.0 | 2 | 1,500 | H, B, G |
| A22 | 3.5 y | 0.1 | 2 | 2,500 | B |
| A23 | 3.5 y | 0.1 | 2 | 2,500 | H, B, G |
| A24 | 3.5 y | 0.1 | 2 | 2,500 | H, B, G |
| A25 | 11.5 y | 1.0 | 2 | 8,000 | H, B, G |
| A26 | 8.5 y | 0.1 | 2 | 13,000 | B |
| A27 | 2.5 y | 1.0 | 1 | 1,500 | H, B, G |
| A28 | 2.5 y | 0.1 | 1 | 2,500 | H, B, G |
| B1 | 8 y | 1.0 | 1.6 | 4,400 | C, U |
| B2 | 8 y | 0.1 | 1.33 | 3,850 | C, BC |
| B3 | 1 y | 1.0 | 0.57 | 22 | H, B, BC |
| B4 | 10 y | 0.1 | nd | nd | BC |
| B5 | 10 y | 0.1 | nd | 3,500 | H, B, G, BC |
| B6 | 10 y | 1.0 | nd | 4,500 | H, B, G, BC, U |
| B7 | 10 y | 1.0 | 1.7 | 4,400 | H, B |
| B8 | 6 y | 0.1 | 2 | 5,000 | H, B |
| B9 | 5 y | 1.0 | 2.65 | 14,400 | H, B, G, C |
| B10 | 7 y | 1.0 | 1.5 | 1,250 | H, B, G, C |
| B11 | 0.5 y | 1.0 | 0.58 | 120 | H, B, G, C |
| B13 | 20 y | 1.0 | 1.66 | 3,700 | H, B, G |
| B14 | 3 mo | nd | 0.86 | 91 | H, B, G |
| B15 | 3 mo | nd | 0.83 | 97 | H, B, G |
| B16 | 3 mo | nd | 0.8 | 120 | H, B, G |
| B17 | 3 mo | nd | 0.58 | 84 | H, G |
| B18 | 4 mo | nd | 0.6 | 82 | H, B, G, C |
| B19 | 4 mo | nd | 0.57 | 74 | H, B, G |
| B20 | 4 mo | nd | 0.63 | 50 | B, C |
| B21 | 4 mo | nd | 0.6 | 90 | B, C |
| C1 | 5 y | 0.1 | 2 | 10,000 | B, C |
| C2 | 5 y | 1.0 | 1.2 | 3,000 | B, C |
| C3 | 2 y | 0.1 | 1.3 | 3,000 | B, C |
| C4 | 5 y | 1.0 | 0.7 | 600 | C |
| C5 | 4 y | 1.0 | 0.7 | 600 | B, C |
| C6 | 2 y | 0.1 | 1 | 700 | C |
| C7 | 2 y | 1.0 | 0.5 | 100 | B, C |
| D1 | 6 y | 1.0 | 1.8 | 4,500 | B, C |
| D2 | 9 y | 0.1 | 2.4 | 1,700 | B, C |
| D3 | 1 y | 0.1 | 0.9 | 500 | H, B |
| D4 | 1 y | 0.1 | 0.9 | 500 | H |
| D5 | 1 y | 1.0 | 0.9 | 500 | H, B |
| D6 | 1 y | 1.0 | 0.9 | 500 | B |
| E1 | 6 y | 0.1 | 2.1 | 9,000 | H, B |
| E2 | 5 y | 0.1 | 1.8 | 5,400 | H, B |
| E3 | 6 y | 1.0 | 1.8 | 4,500 | BC |
| E4 | 5 y | 1.0 | 1.7 | 3,320 | H, B |
| E5 | 6 y | 1.0 | 2 | 6,000 | B, G |
| F1 | 4 y | 1.0 | 0.5 | 580 | H, B, G |
| F2 | 8 y | nd | 1.5 | 3,800 | H, B, G |
| F3 | 8 y | 1.0 | 1.2 | 2,800 | H, B, G |
| G1 | 8 y | 1.0 | 1.5 | 820 | H, B |
| G2 | 9 y | 0.1 | 1.8 | 1,780 | H, B, G |
| G3 | 9 y | 0.1 | 1.8 | 1,780 | B |
| H1 | adult | 1.0 | 2 | 4,500 | B |
| H2 | adult | 1.0 | 1.3 | 3,500 | H, B, G |
| H3 | adult | 0.1 | 1.5 | 2,500 | H, B, G |
| I1 | >10 y | 1.0 | 2 | 3,800 | BC |
| I2 | >10 y | 0.1 | 2.3 | 7,200 | BC |
| J | 1.5 y | 0.1 | 0.89 | 360 | B, G |
| K | adult | 0.1 | nd | nd | H, B, G |
| L | 5 y | 0.1 | 1.8 | 3,000 | U |
| M | 5 y | 0.1 | 2.27 | 5,450 | C |

Legend: 1.0 – male; 0.1 – female; nd - not determined; mo – months; y - years.
